# Metabolites with Antioxidant Activity from Marine Macroalgae

**DOI:** 10.3390/antiox10091431

**Published:** 2021-09-08

**Authors:** Leto-Aikaterini Tziveleka, Mohamed A. Tammam, Olga Tzakou, Vassilios Roussis, Efstathia Ioannou

**Affiliations:** 1 Section of Pharmacognosy and Chemistry of Natural Products, Department of Pharmacy, National and Kapodistrian University of Athens, Panepistimiopolis Zografou, 15771 Athens, Greece; ltziveleka@pharm.uoa.gr (L.-A.T.); mtammam@pharm.uoa.gr (M.A.T.); tzakou@pharm.uoa.gr (O.T.); roussis@pharm.uoa.gr (V.R.); 2 Department of Biochemistry, Faculty of Agriculture, Fayoum University, Fayoum 63514, Egypt

**Keywords:** macroalgae, marine metabolites, antioxidant activity, scavenging, reactive oxygen species

## Abstract

Reactive oxygen species (ROS) attack biological molecules, such as lipids, proteins, enzymes, DNA, and RNA, causing cellular and tissue damage. Hence, the disturbance of cellular antioxidant homeostasis can lead to oxidative stress and the onset of a plethora of diseases. Macroalgae, growing in stressful conditions under intense exposure to UV radiation, have developed protective mechanisms and have been recognized as an important source of secondary metabolites and macromolecules with antioxidant activity. In parallel, the fact that many algae can be cultivated in coastal areas ensures the provision of sufficient quantities of fine chemicals and biopolymers for commercial utilization, rendering them a viable source of antioxidants. This review focuses on the progress made concerning the discovery of antioxidant compounds derived from marine macroalgae, covering the literature up to December 2020. The present report presents the antioxidant potential and biogenetic origin of 301 macroalgal metabolites, categorized according to their chemical classes, highlighting the mechanisms of antioxidative action when known.

## 1. Introduction

In all aerobic organisms, oxygen is a crucial element in their metabolic pathways. A high redox potential milieu stimulates the production of free radicals, defined as chemical species with unpaired valence electrons [1]. The most common reactive species in biological systems are oxygen radicals or oxygen-derived species, such as superoxide anion (O_2_^−^), hydrogen peroxide (H_2_O_2_), and hydroxyl radicals (·OH) [2,3], collectively named reactive oxygen species (ROS). Still, other forms of radicals, such as nitric oxide (NO·) and transition metal ions, can also be produced. ROS are generated as products of normal cellular functioning and oxygen metabolism and have essential functions in various important biochemical processes, such as the defense against infections, vasodilation, neurotransmission, gene regulation, and oxidative signaling [3,4].

**Figure 1:**
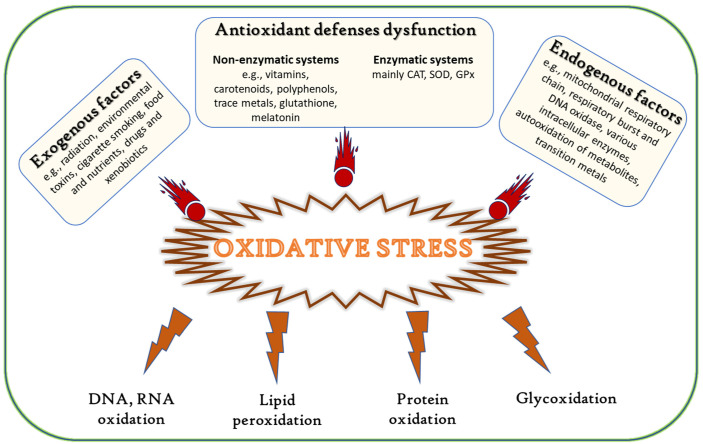
Causes and effects of oxidative stress (adapted from [6]).

Disturbance of the equilibria of prooxidant/antioxidant reactions in cells can lead to redox imbalance and oxidative stress, which causes an excessive generation of ROS and free radicals, in turn resulting in severe cellular damage (Figure 1) [3,5,6,7,8,9]. These molecules further react with key organic substrates, such as DNA, RNA, proteins, and lipids, leading to disruption of their structure or function, and consequently to the onset of diseases, such as atherosclerosis [10], diabetes [11], rheumatoid arthritis [12], inflammatory diseases [13], neurodegenerative diseases [14,15], aging, immune system disorders, and cancer [16,17].

The defense system of living organisms against free radicals comprises both enzymatic and non-enzymatic antioxidants [18]. Enzymes either prevent the formation of or neutralize free radicals (e.g., superoxide dismutases (SOD), catalases (CAT), lactoperoxidases, and glutathione peroxidases (GPx)), or indirectly neutralize free radicals by supporting the activity of other endogenous antioxidants (e.g., glutathione reductase (GR) and glucose-6-phosphate dehydrogenase) [19]. On the other hand, non-enzymatic antioxidants are compounds, other than enzymes, that act on free radicals and can be either produced by the stressed living organism or delivered through the diet, e.g., via the consumption of ascorbic acid (vitamin C), tocopherol (vitamin E), β-carotene, flavonoids, and polyphenols [20]. The most effective and extensively used strategy to diminish oxidative stress is the supplementation of exogenous antioxidants [21]. In recent years, safety and health concerns have been raised for synthetic antioxidants. Therefore, natural antioxidants have attracted attention and are being widely used [1]. Since 2007, antioxidants have been defined as “any substance that delays, prevents or removes oxidative damage to a target molecule’’ [2].

Oceans, covering about 70% of Earth’s surface and hosting an immense array of macro- and microorganisms, constitute a renewable resource of potential therapeutic agents. The diverse and antagonistic marine environment triggers the production of a wide variety of bioactive compounds. Marine organisms have adapted remarkably to extreme environmental conditions, such as high salinity, low or high temperature, high pressure, low availability of nutrients, and low or high exposure to sunlight [22], and can, therefore, provide an outstanding reservoir of bioactive compounds, many of which are unprecedented in terrestrial organisms [23,24,25,26,27].

Marine algae constitute a rich source of structurally diverse natural products, often exhibiting significant biological activities [28,29]. Algae are growing in ecosystems with intense exposure to sunlight and high concentrations of oxygen, conditions that favor the production of free radicals. However, the absence of oxidative damage in structural fatty acid membranes suggests that these organisms synthesize compounds with antioxidant activity [30]. In recent years, several studies highlight the antioxidant potential of seaweeds, attributed to natural products belonging to different structural classes [31,32,33,34,35,36].

A high number of compounds isolated from green, brown, and red algae (Chlorophyta, Ochrophyta, and Rhodophyta, respectively) have been proven to exert prominent antioxidant activity. This review compiles the progress made concerning the discovery of antioxidant compounds derived from marine macroalgae, covering the literature up to December 2020. Following a brief overview of the most commonly used methods for the evaluation of antioxidant activity, algal metabolites with antioxidant activity are presented according to their chemical classification in five main groups, namely (1) phenolic compounds, including bromophenols, phlorotannins, and flavonoids, (2) terpenoids, including steroids & carotenoids, (3) meroterpenoids, (4) nitrogenous compounds, including peptides, alkaloids and chlorophyll-related pigments, and (5) carbohydrates and polysaccharides. Their structural characteristics, the assays used to evaluate their activity, and the measured antioxidant activity levels (when reported in numerical form) are presented, while the mechanisms of antioxidative action are discussed when known.

## 2. Brief Overview of the Methods Employed for the Evaluation of Antioxidant Activity

Efficient antioxidants typically have high redox potential that allows them to act as reducing agents, hydrogen donors, or singlet oxygen quenchers. There are many techniques for evaluating the antioxidant activity, including free radical scavenging, oxygen scavenging, singlet oxygen quenching, metal chelation and inhibition of oxidative enzymes [37]. Overall, in vitro antioxidant tests using free radical traps are relatively straightforward to perform. However, antioxidant activity cannot be securely proposed based on the results from a single assay due to the differences observed between the various test systems [38]. Huang et al. (2005) roughly classified the most important antioxidant capacity assays, according to the reactions involved, into two types: (a) the hydrogen atom transfer (HAT)-based reactions which quantify hydrogen atom donating capacity, and (b) the electron transfer (ET)-based reactions which measure the reducing capacity of antioxidants [39] (Table 1). In HAT-based assays, the antioxidant and the substrate compete for peroxyl radicals. The most commonly used HAT-based assays include the oxygen radical absorbance capacity (ORAC) [40] and the total radical trapping antioxidant potential (TRAP) [41] assays. On the other hand, in ET-based assays the capacity of an antioxidant to reduce an oxidant is measured. The most common ET-based assays include the determination of the total phenolics content (TPC) using the Folin–Ciocalteu reagent [42], the trolox equivalence antioxidant capacity (TEAC)/2,2-azino-bis(3-ethyl benzothiazoline-6-sulfonic acid) diammonium salt (ABTS^+^) radical scavenging [43], the ferric reducing antioxidant power (FRAP) [44,45], and 1,1-diphenyl-2-picrylhydrazyl (DPPH) [46,47] assays.

Antioxidant activity evaluation can also be performed in vivo in animal models, such as in Wistar rats or mice. SOD, CAT, glutathione (GSH), GPx, oxidized low-density lipoprotein (LDL), malondialdehyde (MDA), and GR are the major in vivo indicators of oxidative stress that are usually monitored [48,49].

## 3. Phenolic Compounds

**Figure 2:**
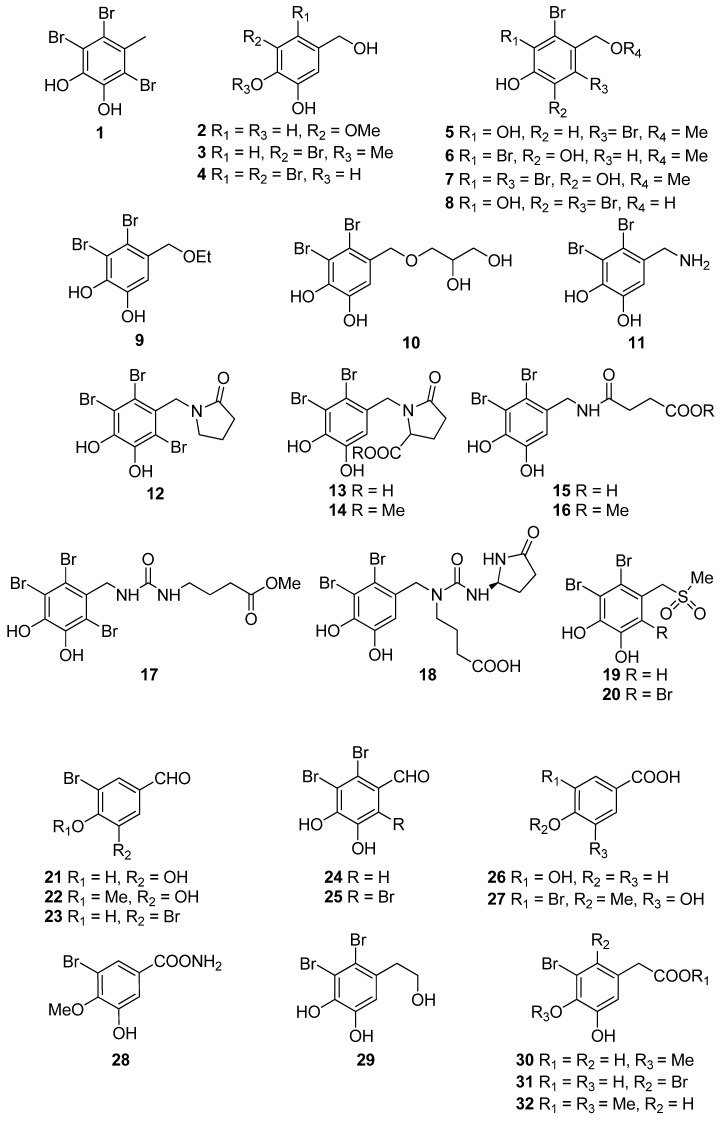
Chemical structures of compounds **1**–**32**.

**Figure 3:**
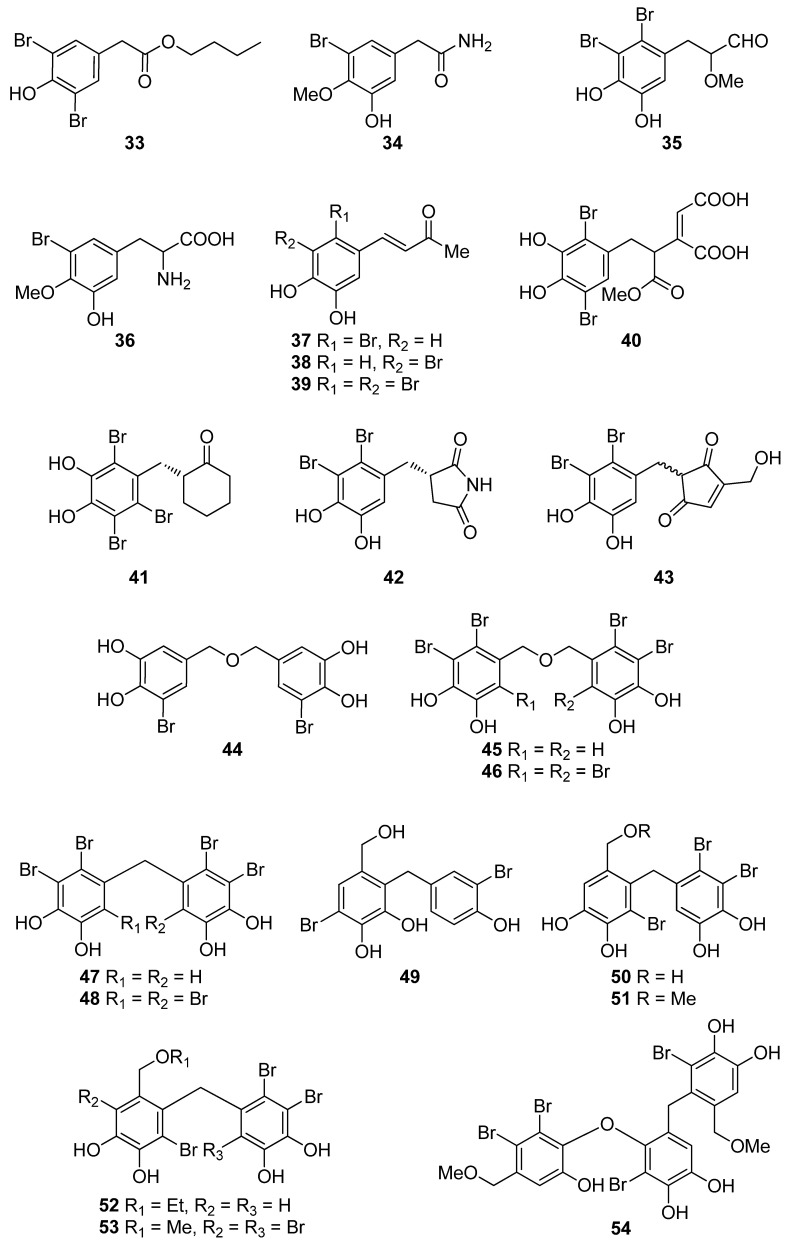
Chemical structures of compounds **33**–**54**.

**Figure 4:**
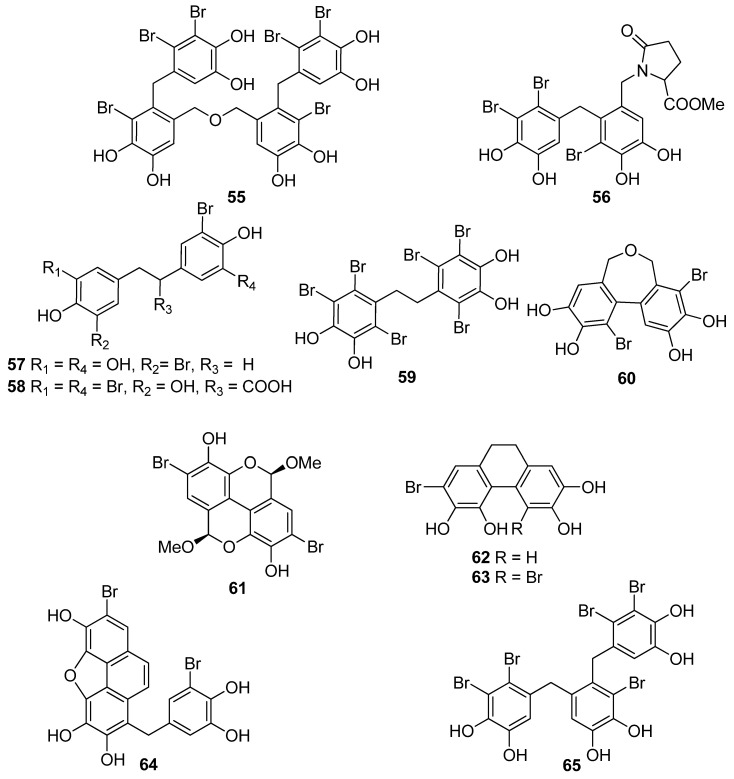
Chemical structures of compounds **55**–**65**.

**Figure 5:**
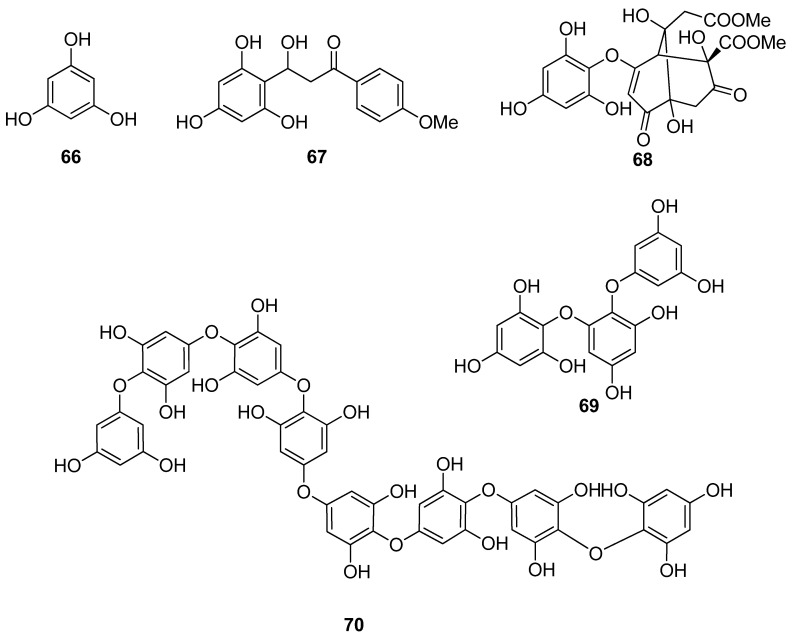
Chemical structures of compounds **66**–**70**.

**Figure 6:**
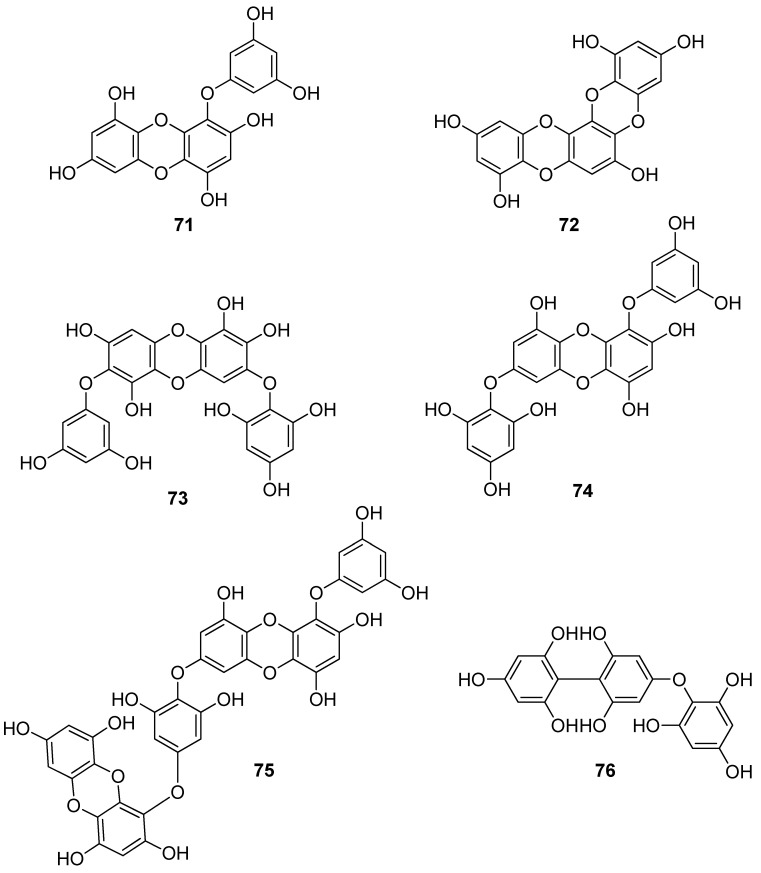
Chemical structures of compounds **71**–**76**.

**Figure 7:**
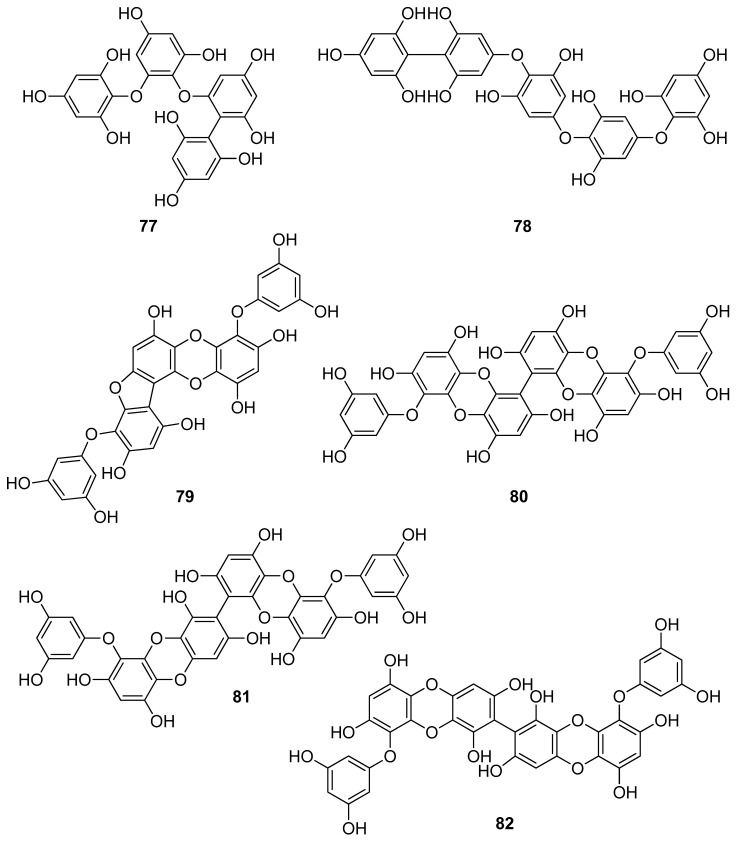
Chemical structures of compounds **77**–**82**.

**Figure 8:**
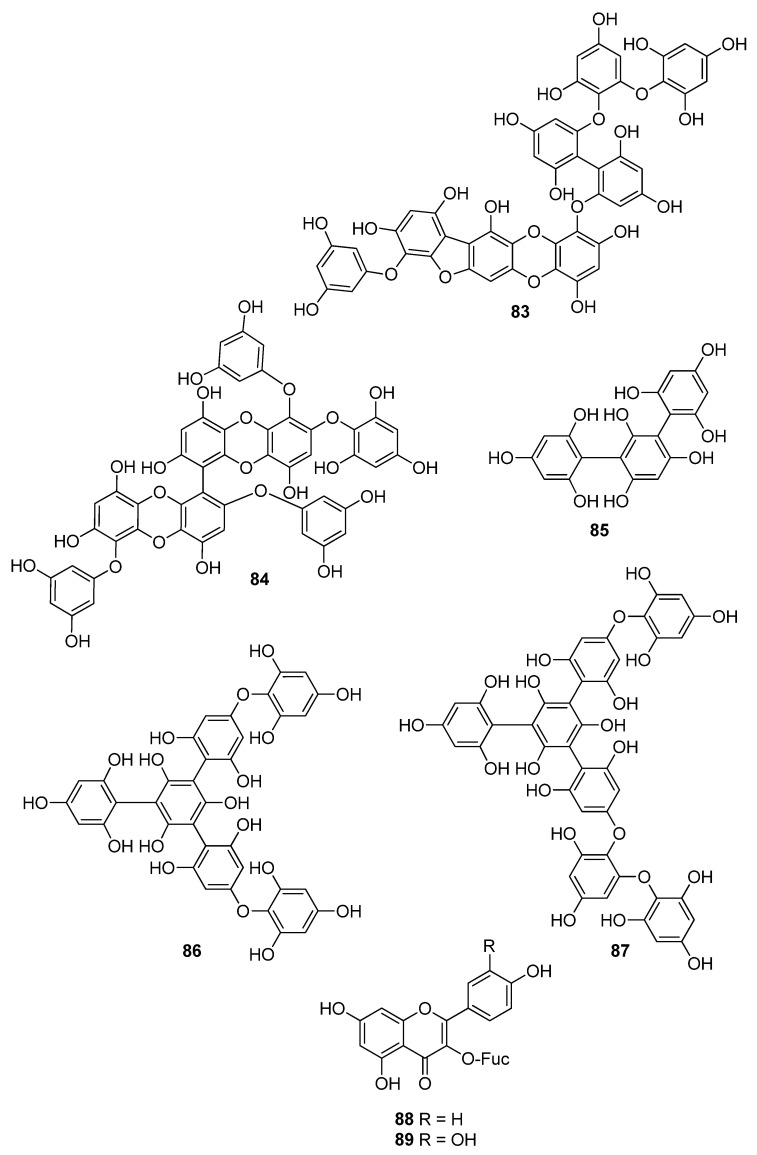
Chemical structures of compounds **83**–**89**.

Phenols comprise a class of chemical compounds containing an aromatic ring bearing a hydroxyl group. Phenolic compounds are classified either as simple phenols or polyphenols based on the number of phenol units in their molecule. Bromophenols (BPs) are marine secondary metabolites containing one or several phenols with one or more bromine atoms in their molecule. Many BPs have been isolated and identified from a variety of marine species, including red, brown, and green algae, as well as ascidians and sponges [73]. Phlorotannins constitute another important and diverse group of naturally occurring polyphenolic secondary metabolites, restricted though to marine algae. Table 2 presents the phenolic compounds, including BPs, phlorotannins, and flavonoids (Figure 2, Figure 3, Figure 4, Figure 5, Figure 6, Figure 7 and Figure 8), isolated so far from marine macroalgae that exhibit significant antioxidant activities.

Recent studies reveal BPs to be one of the most promising candidates in the prevention of diseases associated with free radical attack [73]. Hitherto, more than 60 BPs, mainly isolated from marine red algae, have been reported to exert antioxidant activity in vitro. Their antioxidant activity has been primarily determined by the DPPH radical scavenging method. In general, the BPs shown in Table 2 exhibited better activity than that of butylated hydroxytoluene (BHT, IC_50_ = 82.1 µM), a synthetic antioxidant often used as positive control, with BPs isolated from the red algae *Polysiphonia urceolata*, *Rhodomela confervoides* and *Symphyocladia latiuscula*, as well as the green alga *Avrainvillea* sp. possessing the highest activities in the DPPH assay (IC_50_ < 10.0 µM).

Previous studies have shown that the non-brominated phenolic compounds 5-(hydroxylmethyl)-2-methoxybenzene-1,3-diol (**2**) and 3,4-dihydroxy-benzoic acid (DBA, **26**) exert antioxidant activity [75,77,87]. Specifically, DBA (**26**) was found to reduce the levels of intracellular ROS generated by H_2_O_2_ or UVB treatment of the human HaCaT keratinocytes cells, thus protecting the cells from UVB-induced oxidative stress [87].

A series of BPs (**3**, **4**, **6**, **9**–**11**, **13**–**16**, **18**, **19**, **21**, **22**, **24**, **27**–**32**, **34**–**39**, **42**, **45**, **47**, **50–52**, **56**, **65**) have been isolated from the red alga *R. confervoides* [76,77,81,83,85]. Among them, compounds **10**, **15**, **16**, **18**, **19**, **37**, **38**, **39**, **42**, and **65** exerted the highest antioxidant activity with IC_50_ values of 7.43, 5.43, 5.70, 3.82, 9.52, 8.72, 9.40, 7.62, 5.22, and 8.90 µM, respectively, with all but **37** and **38** bearing a 2,3-dibromo-4,5-dihydroxy-benzyl skeleton. In particular, rhodomelin A (**18**) displayed the highest scavenging activity towards DPPH radical with an IC_50_ value of 3.82 µM. The 2,3-dibromo-4,5-dihydroxy-benzyl unit appears to be a structural element positively influencing antioxidant activity. Moreover, it seems that the antioxidant activity may have a close connection to the number of hydroxyl groups in the molecule [74]. Additionally, the presence of two successive hydroxyl groups in the benzene ring appears to be necessary for increased levels of antioxidant activity to be displayed. This conclusion is supported by the higher IC_50_ values exerted by BPs lacking the second free hydroxyl group by having a methoxyl group instead (e.g., **3**, **22**, **27**, and **36** with IC_50_ values of 50.6, 58.2, 50.3 and 50.9 µM, respectively). For example, compound **22**, with an IC_50_ value of 58.2 µM, bearing only one hydroxyl substituent, is less active than **21** (IC_50_ = 20.3 µM) that possesses the characteristic 4,5-dihydroxy-benzyl group [84,88]. On the other hand, a short and hydrophilic side chain leads to lower activities (e.g., compounds **4**, **6**, **9**, and **11** with IC_50_ values of 42.3, 40.5, 38.4, and 22.5 µM, as compared to compounds **10**, **13**, **14**, **15**, **16**, **18**, and **19** with IC_50_ values of 7.43, 12.4, 14.6, 5.43, 5.70, 3.82, and 9.52 µM, respectively).

Ryu et al. (2019) found that 3-bromo-4,5-dihydroxy-benzaldehyde (**21**) protects human keratinocytes from oxidative stress by upregulating extracellular signal-regulated kinase (ERK) and protein kinase B (Akt), which allows nuclear factor erythroid 2-related factor 2 (Nrf2) to induce the transcription of the antioxidant enzyme heme oxygenase (HO-1) [85].

BPs **8** and **41**, as well as the biphenyl BPs **46**, **48**, **53**, and **59**, isolated from the red alga *S. latiuscula*, all being fully substituted, showed particularly high radical scavenging activity, with IC_50_ values of 7.5, 8.5, 8.5, 8.1, 10.5, and 10.2 µM, respectively, significantly higher than that of L-ascorbic acid (IC_50_ = 15.3 µM), employed as positive control [74,79]. The structurally similar avrainvilleol (**49**), isolated from the green alga *Avrainvillea* sp., also exerted high antioxidant activity with an IC_50_ value of 6.1 µM [91]. The DPPH radical-scavenging activities of the bis-phenols **46**, **48**, **53**, and **59** are noticeably higher than those of the mono-phenols **1**, **7**, **8**, **12**, **20**, **25**, and **41** with IC_50_ values of 14.0, 15.5, 7.5, 14.4, 24.0, 24.7, and 8.5 µM, respectively. Apparently, DPPH scavenging activity is directly related to the overall number of phenol units in the molecules (e.g., **45** and **47** vs. **65** and **14** vs. **56**, with IC_50_ values of 17.6 and 16.9 vs. 8.90, and 18.5 vs. 13.6 μM, respectively). Compounds having the same number of phenolic hydroxyl groups, such as compounds **28** and **34**, or **45** and **47** exhibit similar DPPH radical scavenging activity (23.6 and 20.8, or 17.6 and 16.9 μM, respectively) [76,81].

Furthermore, a series of BPs isolated from the red alga *P. urceolata* (**23**, **33**, **38**, **57**, **58**, **60**–**64**) was shown to exhibit significant DPPH radical scavenging activity [84,88,92]. Among them, compounds **60**, **62**, **63**, and **64**, bearing four hydroxyl groups in their molecules, were the most active with IC_50_ values of 8.1, 6.8, 6.1, and 7.9 µM, respectively. Moreover, in this case, the necessity for the presence of two successive hydroxyl groups in the benzene ring is evident for the display of enhanced antioxidant activity. Another important factor for enhanced activity is the conjugation of the benzene rings, as evidenced by comparing compounds **57** and **63**. The conjugation in the dihydrophenanthrene skeleton results to a reduction in the IC_50_ values from 19.6 µM for **57** to 6.1 µM for **63**.

The degree of bromination does not appear to affect the antioxidant activity in a consistent manner. For example, in the case of BPs **12** and **13** the IC_50_ values were comparable (14.4 and 12.4 µM, respectively). In the case of **19** and **20** (IC_50_ values 9.52 and 24.0 μM, respectively), it appears that the extra bromine atom in **20** reduces the antioxidant activity, while in the cases of **24** and **25**, **45**, and **46**, as well as **47** and **48** it appears that the presence of an additional bromine atom increases the activity. Moreover, by comparing the IC_50_ values of **37** and **38** (8.72 and 9.40 µM, respectively), it appears that the site of bromination is of no decisive importance.

Choi et al. (2018) showed that bis (3-bromo-4,5-dihydroxybenzyl) ether (BDDE, **44**), isolated from *Polysiphonia morrowii*, suppresses the lipopolysaccharide (LPS)-induced ROS generation in RAW 264.7 macrophage cells. In turn, inhibition of LPS-induced ROS generation by BDDE (**44**) caused ERK inactivation and an inflammatory reaction [90]. Therefore, BBDE (**44**) inhibits LPS-induced inflammation by inhibiting the ROS-mediated ERK signaling pathway in RAW 264.7 macrophage cells and thus can be useful for the treatment of inflammatory diseases [90].

Phlorotannins, exclusively found in macroalgae, are oligomers or polymers of phloroglucinol (1,3,5-trihydroxybenzene, PGU, **66**) that can be classified according to the linkage of PGU units [125,126]. Park et al. (2019) suggested that PGU (**66**) is able to protect HaCaT keratinocytes against oxidative stress-induced DNA damage and apoptosis through the activation of the Nrf2/HO-1 signaling pathway [96].

Until now, numerous phlorotannins purified from brown seaweeds, especially from *Ecklonia* sp., have been proven to exert antioxidant activities and protective effects against H_2_O_2_-induced cell damage [93,95,104,105,106,108,110]. In particular, eckol (**71**), eckstolonol (**72**), diphlorethohydroxycarmalol (DPHC, **73**), 7-phloroglucinol-eckol (**74**), dieckol (**75**), fucodiphloroethol G (**77**), phlorofucofuroeckol-A (**79**) 6,6′-bieckol (**80**), 6,8′-bieckol (**81**), 8,8′-bieckol (**82**), 974-B (**83**), and 2,7″-phloroglucinol-6,6′-bieckol (**84**), isolated from *Eisenia bicyclis, Ecklonia cava*, *Ecklonia stolonifera,* and *Ishige okamurae*, have shown potent antioxidant activity as determined by the DPPH radical scavenging method, with IC_50_ values of 11.5, 8.8, 10.5, 18.6, 6.2, 0.60, 4.7, 8.69, 15.0, 0.86, and 0.51 μM, respectively [95,97,104,108,109,115,122,123]. Among them, fucodiphloroethol G (**77**), compound **83**, and 2,7″-phloroglucinol-6,6′-bieckol (**84**) are the most effective, with IC_50_ values in the nanomolar range [115,122,123].

Eckol (**71**) suppresses the production of intracellular ROS and increases GSH levels in HepG2 cells [103], while dieckol (**75**) induces apoptosis in human hepatocellular carcinoma Hep3B cells via the activation of both death receptor and mitochondrial-dependent pathways, by activating caspases-3, -7, -8, -9, and poly(ADP-ribose) polymerase (PARP) [113]. Moreover, eckol (**71**), phlorofucofuroeckol A (**79**), dieckol (**75**), and 8,8′-bieckol (**82**) have shown potent inhibition of phospholipid peroxidation at a concentration of 1 μM in a liposome system [108]. Lee et al. (2018) showed that both eckol (**71**) and dieckol (**75**) attenuated PM_10_ (particulate matter of less than 10 mm) -induced lipid peroxidation and cytokine expression in human epidermal keratinocytes [107]. Similarly, Zhen et al. (2019) showed that DPHC (**73**) blocked PM_2.5_ (fine particulate matter with a diameter ≤2.5 μm) -induced ROS generation in human keratinocytes [111]. Specifically, DPHC (**73**) protected cells against PM_2.5_-induced DNA damage, endoplasmic reticulum stress, and autophagy, and inhibited lipid peroxidation, protein carbonylation, and increased epidermal height in HR-1 hairless mice exposed to PM_2.5_. Moreover, DPHC (**73**) attenuated PM_2.5_-induced apoptosis and mitogen-activated protein kinase (MAPK) protein expression [111]. In the study of Heo et al. (2012), the neuroprotective effect of DPHC (**73**) against H_2_O_2_-induced oxidative stress in murine hippocampal neuronal cells HT22 was investigated and it was found that DPHC protected cells from H_2_O_2_-induced neurotoxicity by restoring cell viability [110]. Specifically, DPHC (**73**) slightly reduced the expression of Bax induced by H_2_O_2_, but recovered the expression of Bcl-xL, as well as caspase-9 and -3 mediated PARP cleavage by H_2_O_2_, while it effectively inhibited intracellular ROS and lipid peroxidation in a dose-dependent manner and suppressed the elevation of H_2_O_2_-induced Ca^2+^ release [110].

On the other hand, the protective effects of 6,6′-bieckol (**80**) against high-glucose-induced oxidative stress were investigated using human umbilical vein endothelial cells (HUVECs) susceptible to oxidative stress [121]. It was found that 6,6′-bieckol (**80**) significantly inhibited the high-glucose treatment-induced HUVECs’ cell death. Moreover, compound **80** dose-dependently decreased thiobarbituric acid reactive substances (TBARS), intracellular ROS generation, and nitric oxide levels that were increased by high glucose. High glucose levels induced the overexpression of inducible nitric oxide synthase (iNOS), cyclooxygenase 2 (COX-2), and nuclear factor-kappa B (NF-κB) proteins in HUVECs, but treatment with 6,6′-bieckol (**80**) reduced their overexpression.

The structure–activity relationship of phlorotannins, although not fully elucidated, suggests that the hydroxyl group availability influences phlorotannins’ antioxidant capacity to a far greater extent than polymerization and the size of the molecule.

Flavonoids are another important class of polyphenolic secondary metabolites often exhibiting potent antioxidant activity, found predominantly in plants and fungi, but also to a lesser degree in algae. The flavonoids acanthophorin A (**88**) and acanthophorin B (**89**), isolated from the red alga *Acanthophora spicifera*, were shown to exert significant antioxidant activity by preventing lipid peroxidation and inhibiting the generation of MDA in liver homogenates of rat in vitro. Compounds **88** and **89**, with IC_50_ values 1.0 × 10^−2^ and 1.5 × 10^−2^ µM, respectively, displayed almost 10,000 times higher activity than vitamin E (IC_50_ = 160 µM) [124].

## 4. Terpenoids

**Figure 9:**
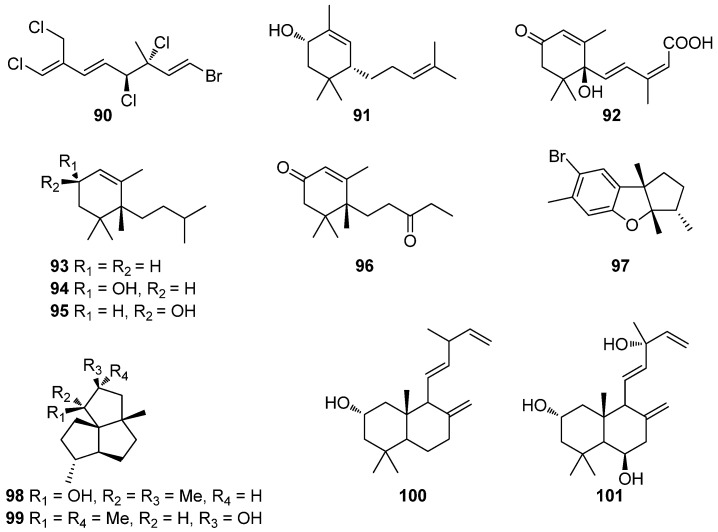
Chemical structures of compounds **90**–**101**.

**Figure 10:**
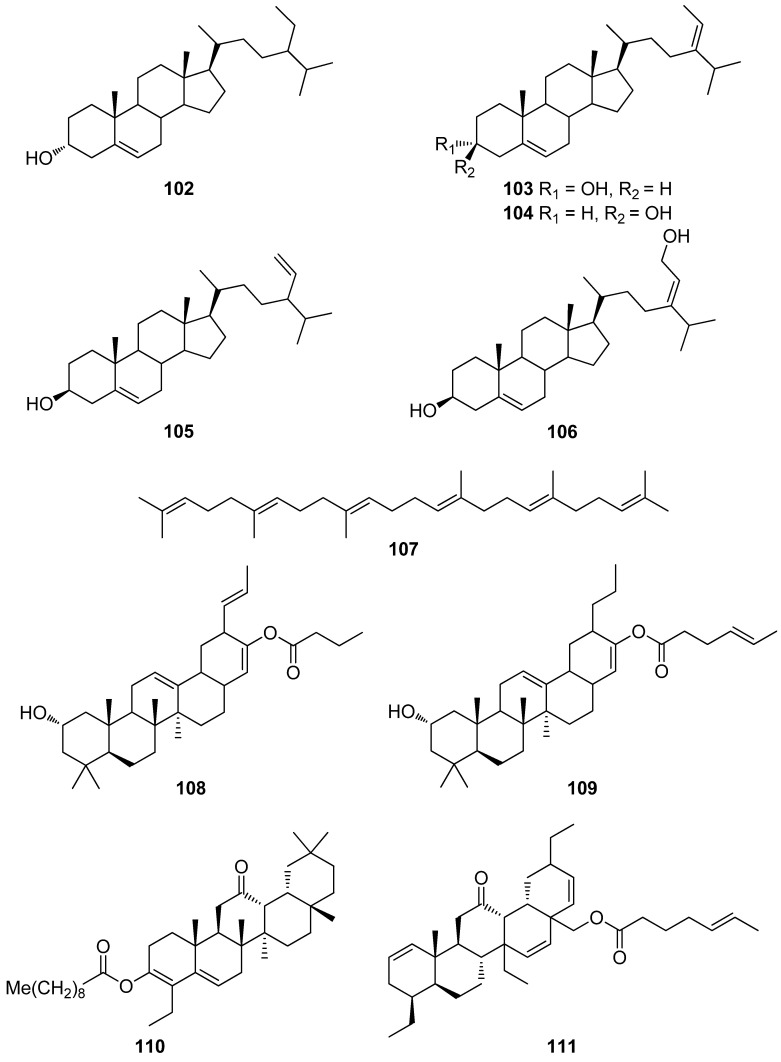
Chemical structures of compounds **102**–**111**.

**Figure 11:**
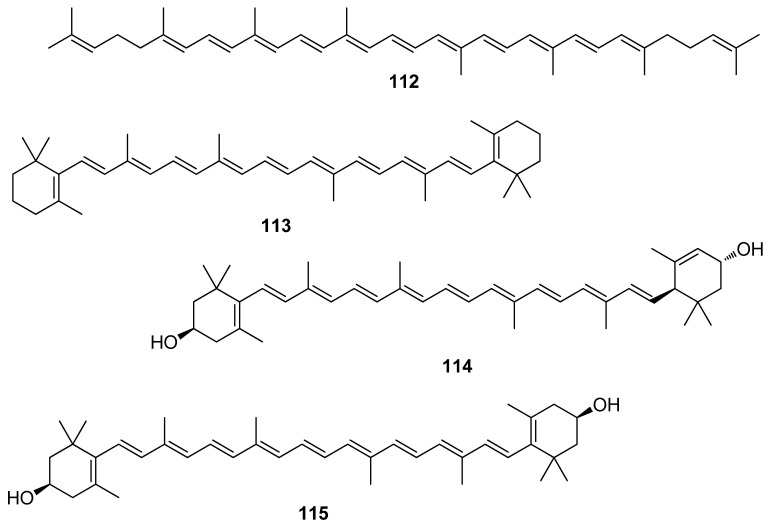
Chemical structures of compounds **112**–**115**.

**Figure 12:**
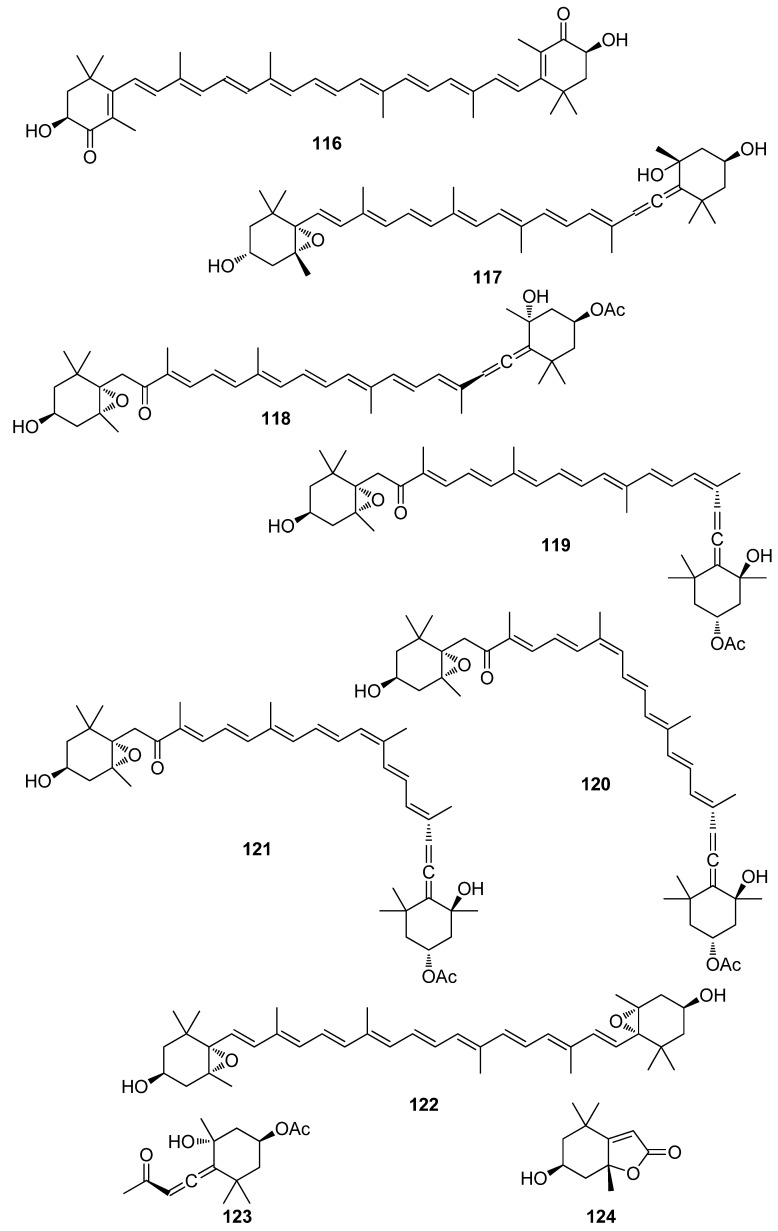
Chemical structures of compounds **116**–**124**.

Terpenoids, also called isoprenoids, represent a diverse class of naturally occurring secondary metabolites composed of isoprene units. Terpenoids, often possessing multicyclic structures with various functional groups [127], are ubiquitous, found in almost all classes of living organisms, including macroalgae. Table 3 presents the terpenoids possessing significant antioxidant activities isolated so far from marine macroalgae (Figure 9, Figure 10, Figure 11 and Figure 12).

Compared to phenolic compounds, as presented in Table 2, it is evident that terpenoids are less active, since their IC_50_ values in the DPPH radical scavenging assay are mostly within the mM range. The most active compounds reported are the halogenated monoterpene (1*E*,3*R*,4*S*,5*E*,7*Z*)-1-bromo-3,4,8-trichloro-7-(dichloro-methyl)-3-methyl- octa-1,5,7-triene (**90**), isolated from the red alga *Plocamium* sp., and the carotenoids fucoxanthin (**118**) and violaxanthin (**122**), isolated from various macroalgae, with IC_50_ values of 50.0, 19.6, and 68.9 μM, respectively [128,159,163].

Alarif et al. (2015) isolated a series of C-29 steroids (**102**–**106**), along with fucoxanthin (**118**), from the brown alga *Cystoseira trinodis* and all compounds were evaluated for their antioxidant activity [135]. Steroids **102**–**106** showed moderate antioxidant activity (20.4 to 27.5%) in the ABST assay, while compound **118** exhibited significant levels of activity (72.1%).

Fucosterol (**104**), frequently isolated from brown algae, was confirmed to exert antioxidant activity on hepatic cells via an increase in the hepatic levels of GSH and a decrease in ROS production, therefore preventing hepatic damage and the resultant increase in alanine transaminase and aspartate transaminase activities [136]. Hence, fucosterol is considered an effective hepatoprotective agent that could be useful for preventive therapies against oxidative stress-related hepatotoxicity.

Moreover, the *abeo*-oleanenes **110** and **111** were isolated from the red alga *Gracilaria salicornia* and their antioxidant activity was evaluated employing the DPPH and ABTS^+^ radical scavenging assays [138]. Compound **110** exhibited higher radical scavenging activities (DPPH IC_50_ = 1.33 mM; ABTS^+^ IC_50_ = 1.09 mM), when compared to those displayed by compound **111** (DPPH IC_50_ = 1.56 mM; ABTS^+^ IC_50_ = 1.24 mM) and α-tocopherol that was used as positive control (DPPH IC_50_ = 1.46 mM; ABTS^+^ IC_50_ = 1.72 mM).

Among terpenoids, carotenoids, a family of lipophilic pigments synthesized by plants, algae, fungi, and microorganisms, but not animals, exhibit high levels of antioxidant activity. In red, brown, and green algae, carotenoids play a key role in their protection against photo-oxidative processes [6]. Their antioxidant action is based on their singlet oxygen quenching properties and their free radicals scavenging ability, which mainly depends on the number of conjugated double bonds, the nature of substituents and the end groups of the carotenoids [6].

In marine macroalgae, β-carotene (**113**), lutein (**114**), zeaxanthin (**115**), astaxanthin (**116**), neoxanthin (**117**), fucoxanthin (**118**), and violaxanthin (**122**) are known to be among the major carotenoids encountered [167]. Astaxanthin (**116**) acts as a safeguard against oxidative damage through various mechanisms, such as singlet oxygen quenching, radical scavenging, inhibition of lipid peroxidation, and regulation of gene expression related to oxidative stress [144,148,168,169,170,171]. The exact mechanisms of action of astaxanthin have been extensively studied, since it has been proven to confer protective effects against neurological diseases, as well as in treating and preventing skin diseases [171,172,173].

Specifically, astaxanthin (**116**) activates the phosphatidylinositol 3-kinase (PI3K)/Akt and ERK signaling pathways, and thus facilitates the dissociation and nuclear translocation of Nrf2, which leads to upregulation of the expression of Nrf2-regulated enzymes (e.g., HO-1, NQO-1, and GST-α1) [147]. Astaxanthin (**116**) inhibits the production of intracellular ROS by negatively regulating the Sp1/NR1 signaling pathway [149,150] and modulating the expression of oxidative stress-responsive enzymes, such as HO-1, which is a marker of oxidative stress and a regulatory mechanism involved in cell adaptation against oxidative damage [143]. In addition, astaxanthin activates the Nrf2/HO-1 antioxidant pathway by generating small amounts of ROS [145,146]. In agreement with these studies, Xue et al. (2017) observed that astaxanthin upregulated Nrf2 expression, as well as Nrf2-targeted proteins HO-1 and antioxidative enzymes SOD2, CAT, and GPx1 in irradiated cells [151]. Thus, astaxanthin (**116**) exerts noteworthy antioxidant activities via both direct radical scavenging, and activation of the cellular antioxidant defense system through modulation of the Nrf2 pathway. Furthermore, a recent study in a rat deep-burn model demonstrated astaxanthin’s protective role in early burn-wound progression by controlling ROS-induced oxidative stress. In that case, the regulation of free radical production is due to the influence of xanthine oxidase and the reduced form of nicotinamide adenine dinucleotide phosphate oxidase, both contributing to the generation of ROS [144].

Fucoxanthin (**118**), often isolated from brown algae, is an oxo-carotenoid with an allenic carbon moiety and a 5,6-monoepoxide in its structure, acknowledged as an efficient quencher of singlet oxygen in photooxidation [174,175,176]. The antioxidant activity of fucoxanthin (**118**) is mediated through various mechanisms, such as singlet oxygen quenching, radical scavenging, and inhibition of lipid peroxidation. Fucoxanthin (**118**) has been shown to exert the best in vitro bioactivities among carotenoids in inhibiting overexpression of vascular endothelial growth factor, resisting senescence, improving phagocytic function, and clearing intracellular ROS in retinal pigment epithelium cells, protecting the retina against photoinduced damage [156].

The study of Taira et al. (2017) demonstrated that fucoxanthin (**118**), through the Nrf2 activation, exerts either cytoprotective activity or induction of apoptosis, depending on the concentrations employed [153]. At a low concentration range (1–4 µM), fucoxanthin provides a cytoprotective effect due to its antioxidant activity, as exerted by its peroxyl radical scavenging capacity, involving the antioxidant HO-1 protein expression increase through the activation of the Nrf2/ARE pathway. On the other hand, high concentration (>10µM) treatment of cells induces apoptosis with caspase -3/7 activation during the suppression of anti-apoptotic proteins, such as Bcl-xL and pAkt.

Besides, the cytoprotective effect of fucoxanthin (**118**) has been investigated against H_2_O_2_-induced cell damage [154,158]. It was shown that fucoxanthin effectively inhibited intracellular ROS formation, DNA damage, and apoptosis induced by H_2_O_2_. Finally, the protective effect of fucoxanthin was investigated against UVB-induced cell injury in human fibroblasts and showed significant decrease in intracellular ROS formation and increase in cell survival rate in a dose-dependent manner [155].

Comparative studies of the radical scavenging efficiency of fucoxanthin (**118**) and its stereoisomers (**119**–**121**) isolated from *Laminaria japonica* have also been conducted [162]. All three stereoisomers had stronger hydroxyl radical scavenging activities than α-tocopherol but showed weaker scavenging activities toward DPPH and superoxide radical, while their radical scavenging activities were not remarkably different, indicating that the differences in the geometry of the double bonds had very little effect on their activity.

Recently, the monoterpenoid (−)-loliolide (**124**) was proven to effectively reduce 2,2′-azobis(2-amidinopropane) dihydrochloride (AAPH)-induced ROS, cell death, and lipid peroxidation in Vero cells and zebrafish embryos in a dose-dependent manner [165]. Moreover, a study conducted by Jayawardena et al. (2019) elaborated the anti-inflammatory effect of *Sargassum horneri* ethanolic extract containing (−)-loliolide on LPS-stimulated RAW 264.7 macrophages via suppression of NF-κB and MAPK and reduction of oxidative stress through the Nrf2/HO-1 pathway [166].

## 5. Meroterpenoids

**Figure 13:**
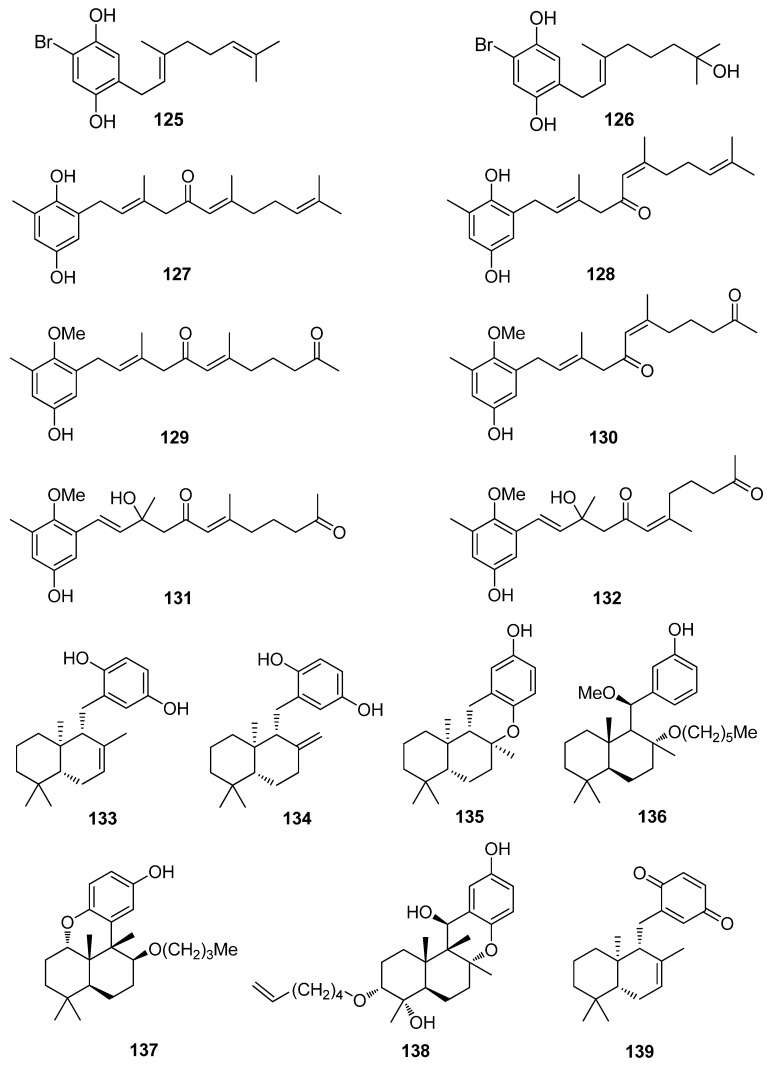
Chemical structures of compounds **125**–**139**.

**Figure 14:**
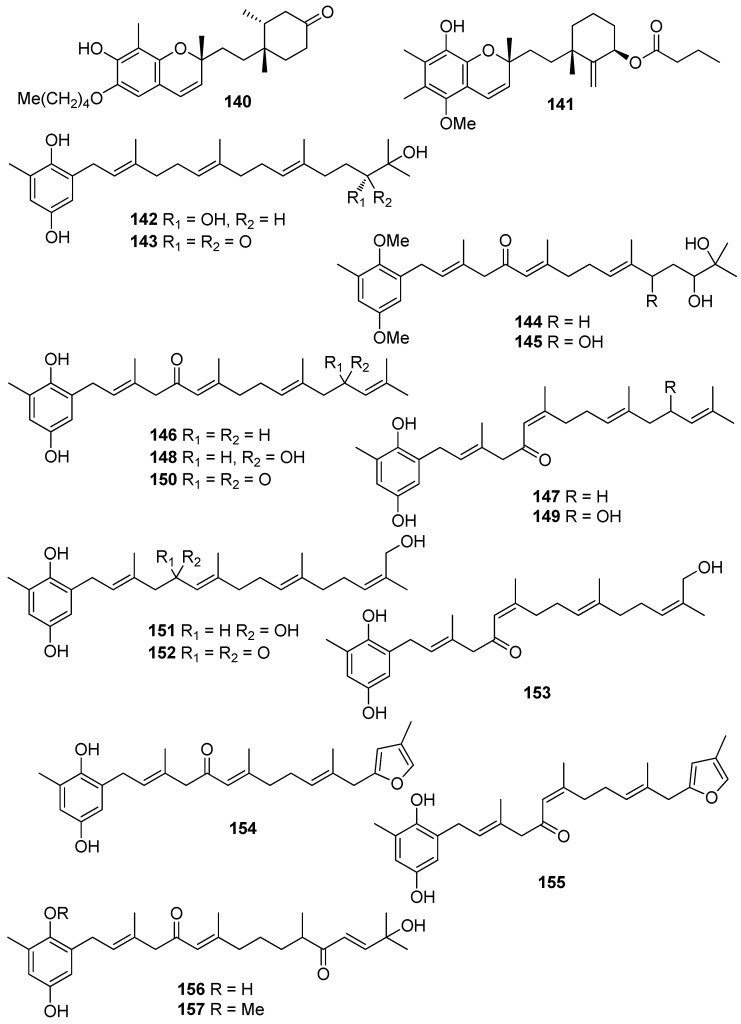
Chemical structures of compounds **140**–**157**.

**Figure 15:**
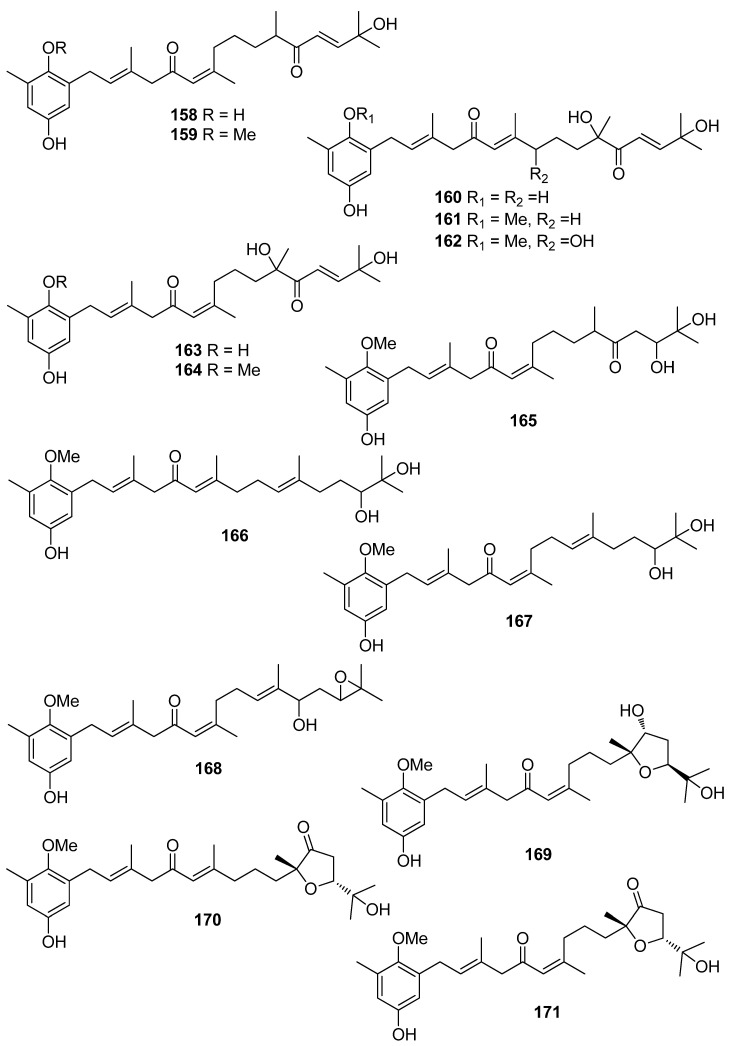
Chemical structures of compounds **158**–**171**.

**Figure 16:**
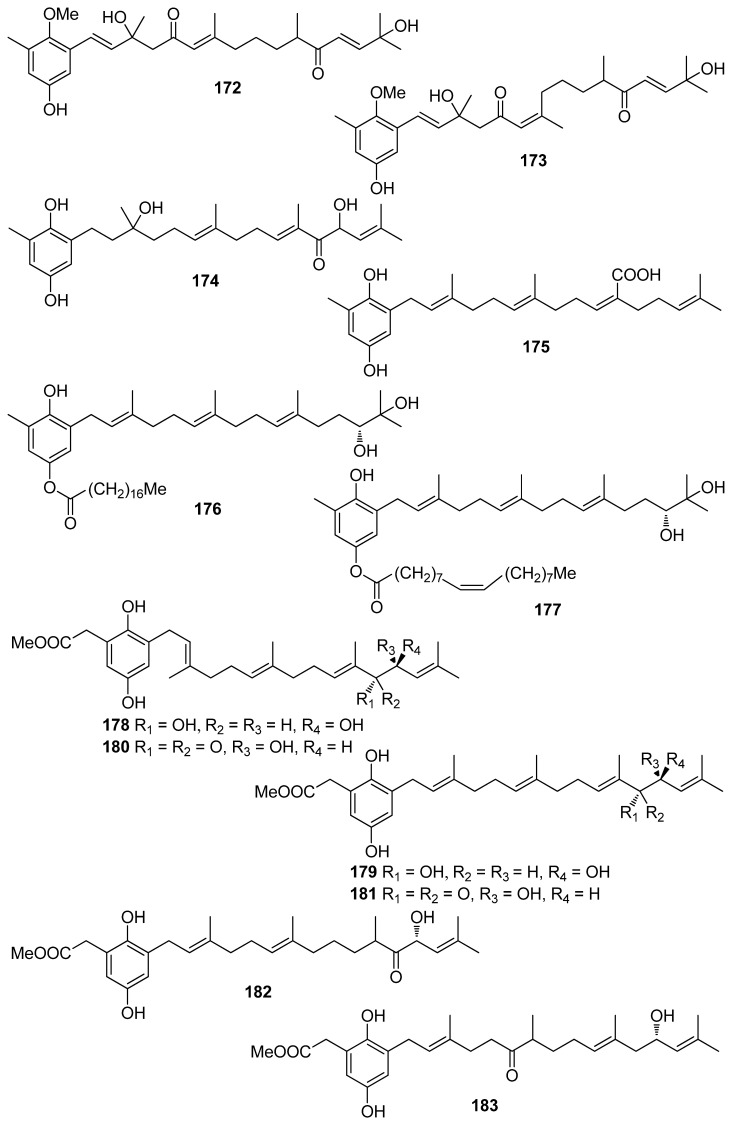
Chemical structures of compounds **172**–**183**.

**Figure 17:**
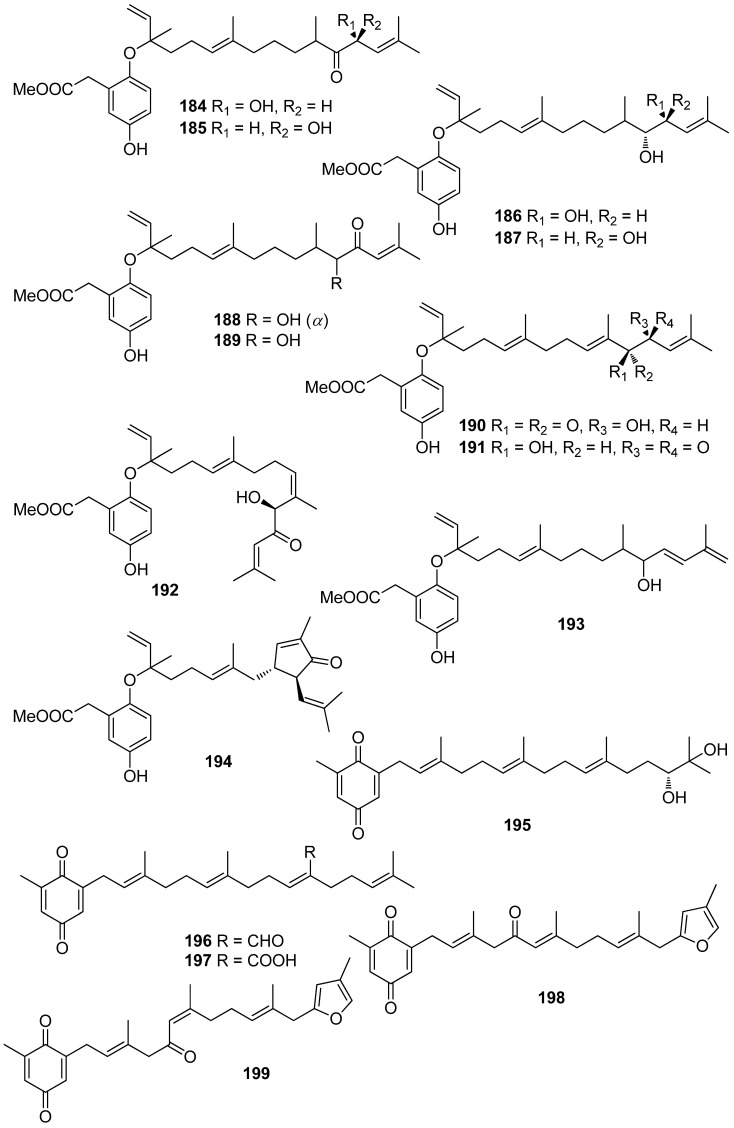
Chemical structures of compounds **184**–**199**.

**Figure 18:**
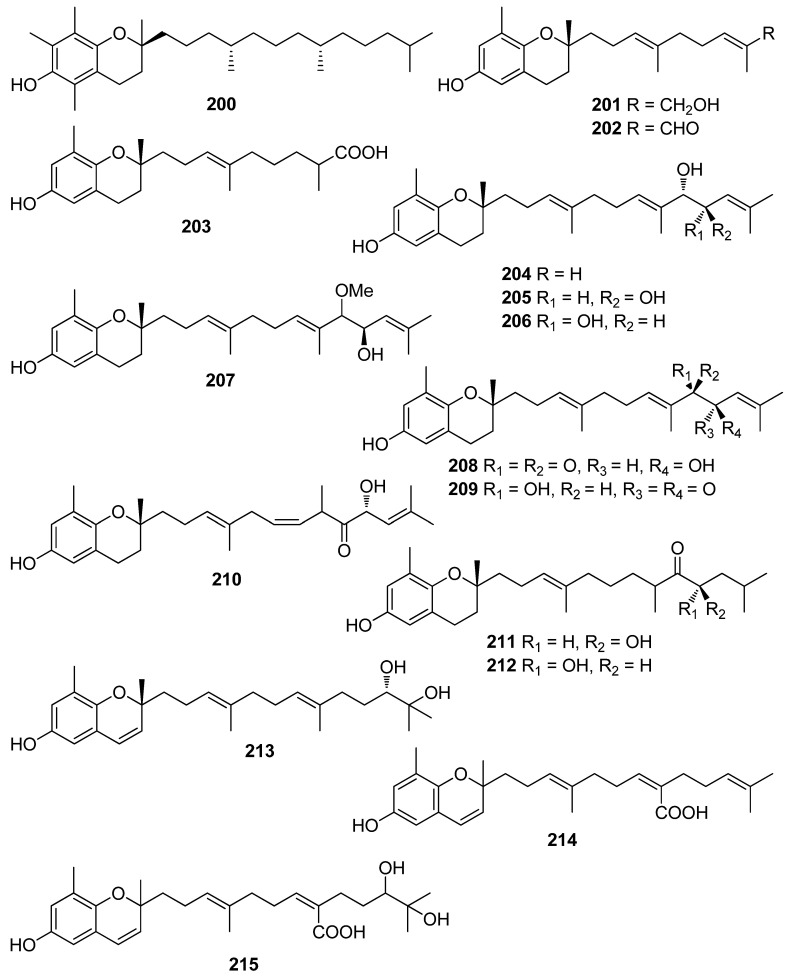
Chemical structures of compounds **200**–**215**.

**Figure 19:**
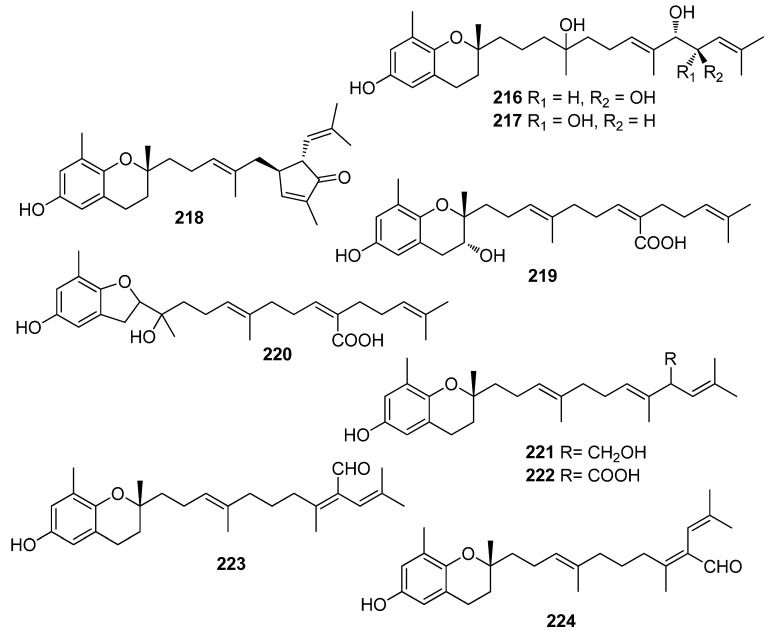
Chemical structures of compounds **216**–**224**.

Meroterpenoids are natural products of mixed biosynthesis containing a terpenoid part that exhibit a variety of biological activities. Metabolites belonging to this class that display antioxidant activity have been isolated from various macroalgae (Table 4, Figure 13, Figure 14, Figure 15, Figure 16, Figure 17, Figure 18 and Figure 19), the majority of which belong to the phylum Ochrophyta, and especially to the genera *Cystoseira* and *Sargassum*.

Overall, meroterpenoids from marine macroalgae have exhibited moderate to remarkable antioxidant activity. Specifically, the brominated compound cymopol (**125**), isolated from the green alga *Cymopolia barbata*, exerted noticeably high DPPH scavenging activity with an IC_50_ value of 4.0 μM [91].

De los Reyes et al. (2013, 2016) described the isolation of meroditerpenoids **129**–**132** and **156**–**173** that have shown radical scavenging activity from the brown alga *Cystoseira usneoides* [178,186]. The most active compounds were cystodiones A (**173**), B (**172**), G (**162**), and H (**158**), cystomexicone B (**129**), amentadione (**156**), amentadione 1′-methyl ether (**157**), 6-*cis*-amentadione 1′-methyl ether (**159**), and 11-hydroxyamentadione (**160**), which exhibited antioxidant activity in the ABTS assay in the range of 77–115% compared to Trolox that was used as a standard.

Additionally, Fisch et al. (2003) reported a number of triprenyltoluquinol derivatives (**127**, **128**, **146**–**155**), isolated from the brown alga *Cystoseira crinita*, that showed very high levels of radical scavenging at a concentration of 230 μM (92.5–96.7% as compared to 95.2% scavenging for α-tocopherol) [177]. In contrast, the co-occurring quinones **197** and **198** showed DPPH radical scavenging activities significantly less than that of α-tocopherol and the hydroquinones, but still comparable to that of BHT, i.e., 29.0% for **197** and 38.6% for **198** as compared to 35.6% scavenging observed for BHT at a concentration of 230 μM. The observed differences in the values obtained in the DPPH assay for the tested compounds were attributed to the existence of small impurities in the samples (e.g., due to autoxidation) and the handling of small amounts rather than to structural variations. On the other hand, in the TBARS assay, potent inhibition of linolenic acid methyl ester peroxidation was observed for all hydroquinones, i.e., 66.5–74.9% inhibition for compounds **127**, **128**, and **146**–**155** at a concentration of 164 μM. These activities were comparable to those of α-tocopherol (72.7%) and BHT (69.3%). Additionally, these compounds showed activities between 13% (**153**) and 59% (**149**) of α-tocopherol in the TEAC test and between 40% (**152**) and 112% (**198**) of α-tocopherol in the PCL assay [177].

Jung et al. (2008) isolated an array of meroterpenoids (**174**, **175**, **178**–**194**) from the brown alga *Sargassum siliquastrum* which exhibited moderate to significant radical scavenging activity in the DPPH assay with IC_50_ values ranging from 0.21 to 47.9 μM (for compounds **183** and **192**, respectively) [187]. The observed more than 200-fold increase in the radical scavenging activity of the isonahocols (**174**, **175**, **178**–**183** with IC_50_ values of 0.54, 0.40, 0.27, 0.25, 0.64, 0.68, 0.62, and 0.21 μM, respectively) in comparison to that of the nahocols (**184**–**194** with IC_50_ values of 23.3, 26.1, 25.4, 37.9, 35.4, 18.7, 25.9, 30.4, 47.9, 26.3, and 25.1 μM, respectively) indicated the pivotal role of the second free hydroxyl group in the phenol ring for enhanced radical scavenging activity. Along this trend, the absence of a free phenolic hydroxyl group resulted in lack of scavenging activity [187].

Another investigation conducted by Jang et al. (2005) reported the isolation of meroterpenoids belonging to the subclasses of chromenes and chromenols (**201**, **202**, **204**–**212**, **218**, **221**–**224**) from the brown alga *S*. *siliquastrum* that exhibited over 87% radical scavenging activity at a concentration of 0.23 to 0.29 mM (0.1 mg/mL) [191]. Moreover, the antioxidant activity of compounds **205**, **206**, and **209**, along with that of **203**, **216**, and **217**, was evaluated in various assays, including scavenging effects on the generation of intracellular ROS, increments of intracellular GSH levels, and inhibitory effects on lipid peroxidation in human fibrosarcoma HT 1080 cells [192]. All tested compounds significantly decreased the generation of intracellular ROS, while increasing the levels of intracellular GSH at a concentration of 5 μg/mL, and inhibited H_2_O_2_-induced lipid peroxidation at a concentration of 50 μg/mL.

In an effort to elucidate the mechanism of antioxidant activity of zonarol (**134**), Shimizu et al. (2015) studied its effect on neuronal cells and proved that zonarol protects them from oxidative stress by activating the Nrf2/ARE pathway and inducing phase-2 enzymes [180].

Moreover, Yoon et al. (2013) elucidated the role of sargachromanol G (**208**), isolated from the brown alga *S. siliquastrum*, in receptor activator of NF-κB ligand (RANKL)-induced osteoclast formation [193]. Compound **208** was found to inhibit RANKL-induced osteoclast differentiation from RAW264.7 cells without signs of cytotoxicity. Additionally, the expression of osteoclastic marker genes, such as tartrate-resistant acid phosphatase (TRAP), cathepsin K (CTSK), matrix metalloproteinase 9 (MMP9), and calcitonin receptor (CTR), was also strongly inhibited. It was concluded that sargachromanol G inhibits RANKL-induced activation of NF-κB by suppressing RANKL-mediated IκB-α protein degradation, and therefore the phosphorylation of mitogen activated protein kinases (p38, JNK, and ERK).

## 6. Nitrogenous Compounds

**Figure 20:**
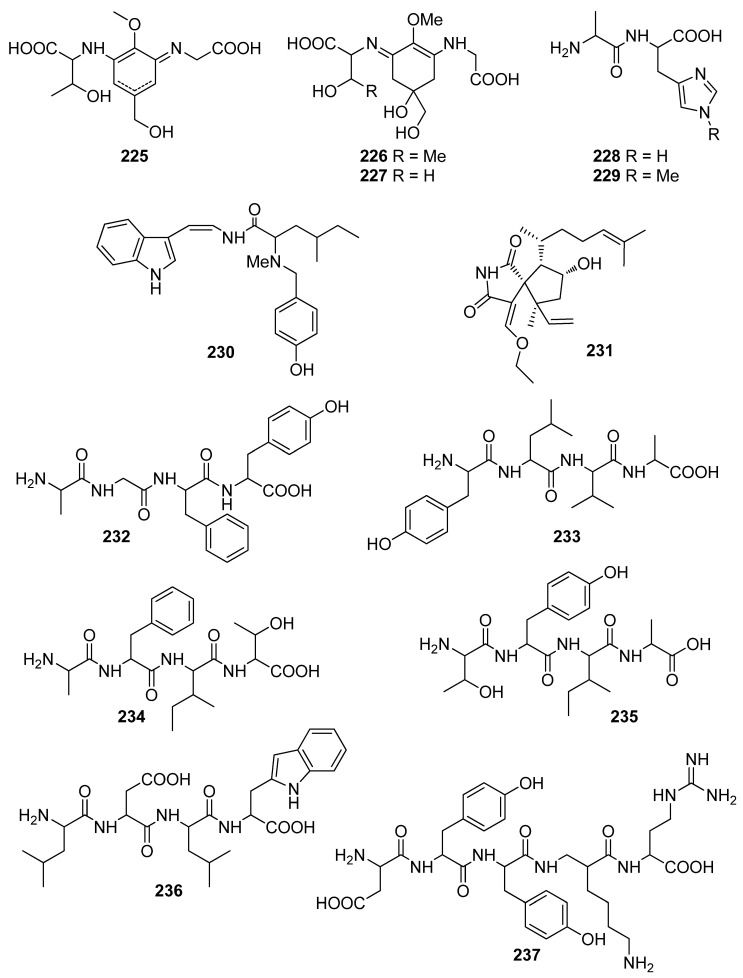
Chemical structures of compounds **225**–**237**.

**Figure 21:**
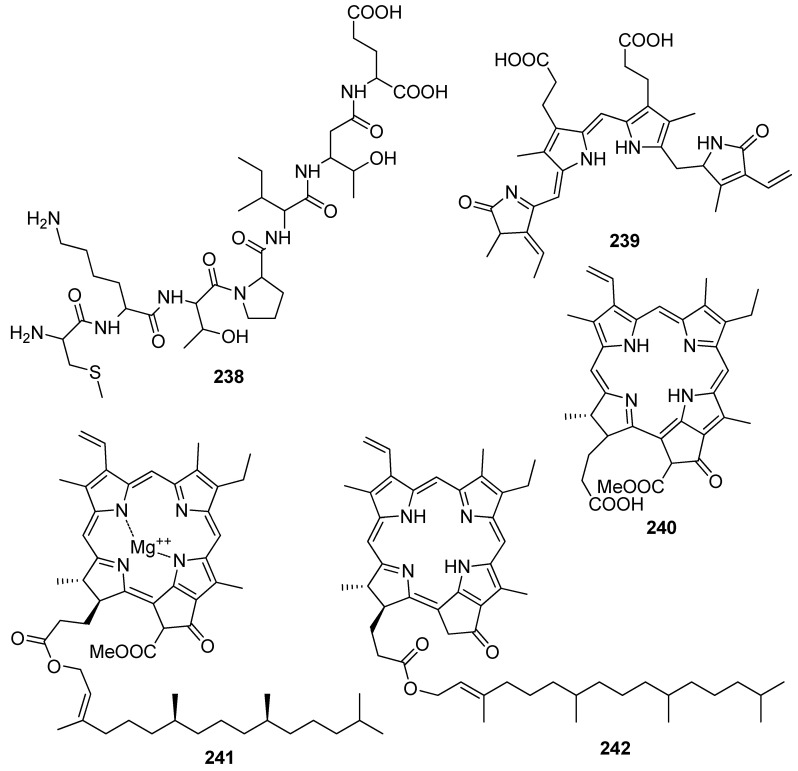
Chemical structures of compounds **238**–**242**.

So far, a number of nitrogenous compounds, including peptides, alkaloids, and chlorophyll-related pigments (Figure 20 and Figure 21), isolated from marine macroalgae have shown antioxidant activity (Table 5).

Peptides and alkaloids **225**–**239**, isolated from *Gloiopeltis furcata*, *Porphyra* sp., and *Martensia fragilis*, have demonstrated moderate to significant antioxidant activity [91,196,197,198,199,200]. Specifically, mycosporine-like amino acids **225**–**227** exhibited markedly lower free radical scavenging activities compared to those of ascorbic acid and Trolox [196,197], although heat treatment of porphyra 334 (**225**) at temperatures over 100 °C afforded its dehydrated form (**227**) and resulted in more than a 100-fold increase in the DPPH radical scavenging activity (IC_50_ =10.1 μg/mL for **227** vs. >1000 μg/mL for **225**) [197].

The histidine-related dipeptides carnosine (**228**) and anserine (**229**) were shown to exert comparable antioxidant activities, as measured by ferric thiocyanate and TBARS (85.2% and 84.1% inhibition for **228** and 94.4% and 89.1% inhibition for **229**, respectively), to those of α-tocopherol (88.2% and 86.7%, respectively) and BHT (99.8% and 98.2%, respectively) [198]. Moreover, Cermeno et al. (2019) isolated a series of bioactive peptides (**232**–**238**) from *Porphyra dioica* that displayed significant antioxidant activity as assessed using the ORAC assay [200]. It appears that peptides containing tyrosine in their structure (compounds **232**, **233**, **235**, and **237**) possessed higher levels of antioxidant activity.

In an effort to elucidate the mechanism of action of dictyospiromide (**231**), neuron-like PC12 cells were treated with H_2_O_2_, and its cytoprotective effect against the induced oxidative damage was evaluated [199]. Treatment with dictyospiromide increased cell survival in a dose-dependent manner and reduced H_2_O_2_-induced lactate dehydrogenase (LDH) production at a concentration as low as 0.5 μM. Additionally, compound **231** was investigated regarding its implication in the Nrf2/ARE signaling pathway, which regulates the expression of genes involved in cellular antioxidant defense. It was found that dictyospiromide (**231**) exhibited a cytoprotective antioxidant effect in PC12 cells that involved activation of the Nrf2/ARE signaling pathway and enhanced expression of HO-1.

Chlorophylls are natural pigments with a well-known antioxidant activity. Although their radical scavenging activities are reported to be low [203], their inhibitory action in lipid peroxidation was found to be 95% at concentrations as low as 100 μM [204]. However, knowledge is limited regarding the yield of chlorophyll metabolites, their absorption and transportation processes, their metabolic pathways, and their precise oxidation mechanisms. At the in vitro level, only few researchers have studied the stability of chlorophylls during digestion and subsequent absorption through intestinal cells. The major outcome is that chlorophylls α and β are transformed into their corresponding pheophorbides and pheophytins and are absorbed at similar rates to those of carotenoids. Further, it has been shown that pheophorbide a is transported at the intestinal level by a protein-mediated mechanism, with scavenger receptor class B type 1 (SR-BI) being a plausible transporter. These results have been confirmed at the in vivo level, using mice as the experimental model, showing a preferential accumulation of pheophorbide in the liver along with multiple other chlorophyll compounds [205].

The characteristic pigments of the light harvesting proteins phycoerythrobilin (**239**), pheophorbide a (**240**), chlorophyll β (**241**) and pyropheophytin α (**242**) have been found to exert antioxidant activity [141,201,202,203,204,205,206]. It seems that the porphyrin ring system is important for the expression of antioxidative activity in the dark. Indeed, phycoerythrobilin (**239**) showed potent antioxidant activity in in vitro experiments and significantly inhibited the release of β-hexosaminidase in rat basophilic leukemia cells [207], suggesting that phycoerythrobilin exhibits anti-inflammatory activity. Pheophorbide a (**240**) demonstrated antioxidant activity (88.6 ± 1.3% DPPH scavenging) higher than that of α-tocopherol, and comparable to that of butylated hydroxyanisol (BHA, 85.3 ± 0.2% DPPH scavenging) at a concentration of 0.1 mg/mL [202], while pyropheophytin α (**242**) demonstrated antioxidant activity higher than that of α-tocopherol [206].

## 7. Carbohydrates and Polysaccharides

**Figure 22:**
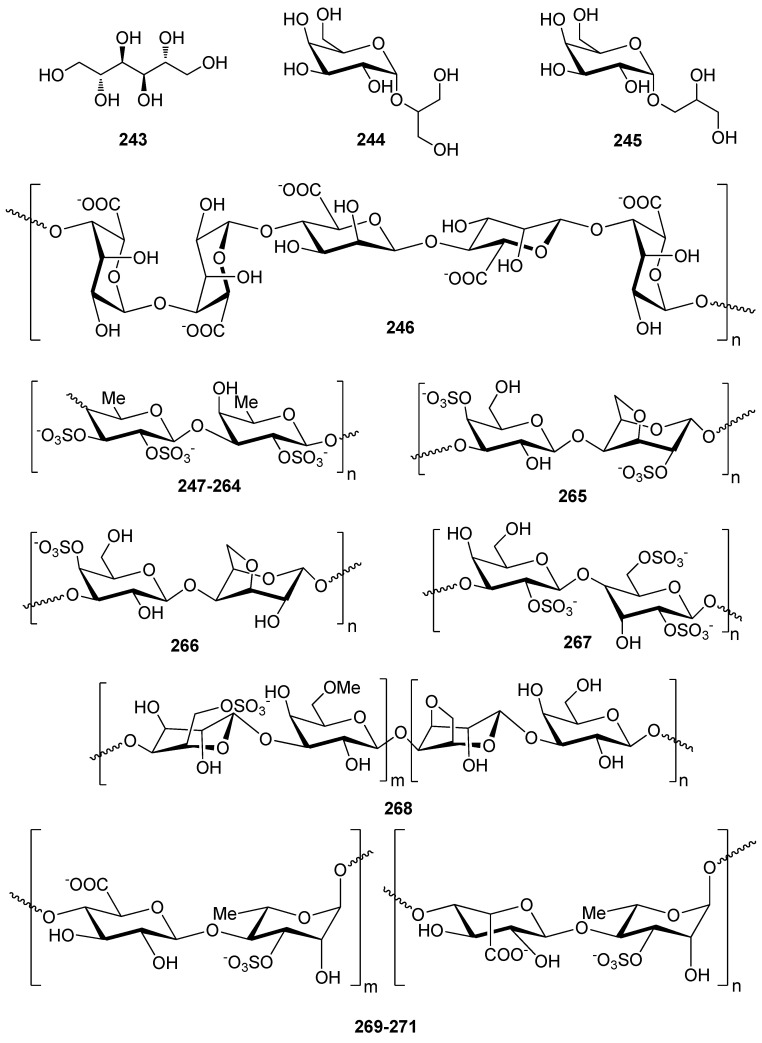
Chemical structures of compounds **243**–**271**.

Carbohydrates ranging in size from simple monosaccharides to high molecular weight polysaccharides isolated from marine macroalgae often exert antioxidant activities [208,209] (Table 6, Figure 22).

The simplest sugar alcohol isolated from a plethora of macroalgae is mannitol (**243**), representing up to 9%, 47%, and 59% of the dry algal weight in Chlorophyta, Rhodophyta and Ochrophyta, respectively [210]. Antioxidant activity evaluation by enzymes (α-glucosidase, acetyl (AChE) and butyrylcholinesterase (BuChE)) and free radicals (DPPH, NO, OH, and O_2_^−^) revealed that higher contents of mannitol are closely related with cholinesterases and DPPH radical scavenging, and to a lesser extent are responsible for α-glucosidase inhibition, OH, O_2_^−^, and NO scavenging.

Two simple glucosides, floridoside (**244**) and D-isofloridoside (**245**), have been isolated from the red alga *Laurencia undulata* and their free radical scavenging activity, inhibition of intracellular ROS levels, the level of membrane protein oxidation, myeloperoxidase (MPO) activity inhibition, gene expression levels of GSH and SOD, and protein expression of MMP2 and MMP9 have been determined [211]. It was found that both floridoside (**244**) and D-isofloridoside (**245**) possess significant antioxidant capacity and are potential inhibitors of MMP2 and MMP9.

Marine macroalgae are the most important source of non-animal sulfated polysaccharides (SPs), with the main categories being fucoidans isolated from brown algae, carrageenans and porphyrans isolated from red algae and ulvans isolated from green algae. SPs possess excellent in vitro antioxidant activity, including both radical scavenging capacity and metal chelating ability [212,227,228]. The antioxidant activity of SPs directly related to their structural features, such as degree of sulfation, molecular weight (MW), type of major sugar, and glycosidic branching [212,225,229]. For example, low MW SPs have shown potent antioxidant activity, stronger than that of high MW SPs [230]. The rationale for this is that low MW SPs may be incorporated into the cells more efficiently and donate proton effectively compared to high MW SPs.

Alginate oligosaccharide (AO, **246**) and fucoidan oligosaccharide (FO, **247**) were enzymatically produced from commercially available polysaccharides and their antioxidant activity was studied [212]. AO (**246**) had the highest hydroxyl radical scavenging activity as compared to FO (**247**), while in the Fe^2+^ chelation assay, FO exhibited good chelation in contrast to AO that hardly displayed any activity.

Fucoidans of diverse MW and sulfation degree (**247**–**264**) have been isolated from various brown algae and/or chemically modified and their antioxidant activity has been tested employing OH and O_2_^−^ scavenging, erythrocyte hemolysis inhibition, metal chelation, and anti-lipid peroxidation assays [212,213,214,215]. In the study of Zhao et al. (2008) two fractions of different MW, namely 742 kDa (**254**) and 175.9 kDa (**255**), were obtained from fucoidans extracted from *L. japonica* and evaluated for their OH and O_2_^−^ scavenging activity, with the higher MW fraction exhibiting higher levels of activity [215]. Following radical process degradation, an ascophyllan-like fraction rich in glucuronic acid and a fraction rich in galactose and mannose were confirmed as responsible for the oxygen free radical scavenging activity [215]. On the contrary, Koh et al. (2019) reported on the higher antioxidant capacity of low MW (10 kDa) fucoidan (**256**) from *Undaria pinnatifida* (close to that of BHA) as compared to a high MW (300 kDa) fucoidan (**257**) [216].

Additionally, Rodriguez-Jasso et al. (2014) isolated fucose-containing sulfated polysaccharides from *Fucus vesiculosus* using either microwave-assisted extraction (**258**) or autohydrolysis (**259**) and their antioxidant activity was determined [217]. Both samples presented similar sulfate contents (~21%), as well as comparable antioxidant potential as evaluated by DPPH and ABTS^+^ scavenging, and lipid oxidation inhibition methods. Differences in the antioxidant potential could be observed only when using a differential pulse voltammetry technique, pointing to structural variations of the fucans obtained by the two different methods.

Several studies have reported the in vitro and in vivo antioxidant efficacy of fucoidan [231]. Kim et al. (2012) have demonstrated that low MW fucoidan (**262**) might block NO, as well as ROS production, suppressing therefore oxidative stress and MAPKs in RAW264.7 cells [220]. Additionally, fucoidan (**263**) was found to reduce the oxidative stress through Nrf2/ERK signaling mediated regulation of HO-1 and SOD1 expression in human keratinocytes [221]. More recently, Phull et al. (2017) have demonstrated that fucoidans derived from *U. pinnatifida* (**264**) exhibit significant in vitro and in vivo anti-arthritic responses in rabbit articular chondrocytes and rats, respectively. Moreover, administration of fucoidan to arthritic rats ameliorated the clinical symptoms and led to the overall improvement of their health [222].

Rocha de Souza et al. (2007) reported on the isolation of iota (ι)- (**265**), kappa (κ)- (**266**), and lambda (λ)- (**267**) carrageenans from various red algae and their antioxidant activity as evaluated by the scavenging of OH and O_2_^−^ radicals, and lipid peroxidation assays [213]. The results of the study indicated that, among the different carrageenans, λ-carrageenan (**267**) exhibited the highest antioxidant and free radical scavenging activity. Thus, a positive correlation between sulfate content and antioxidant activity was evidenced.

Acetylation, phosphorylation and benzoylation of porphyran (**268**) extracted from the red alga *Porphyra haitanensis* afforded derivatives with improved antioxidant activity, as evaluated in superoxide radical, hydroxyl radical and reducing power assays [223]. In a previous study, Zhang et al. (2003) obtained through anion-exchange column chromatography three sulfated polysaccharide fractions with variable sulfate content (17.4%, 20.5% and 33.5%) from the same red algal species and investigated their in vitro antioxidant activities [229]. All three showed strong scavenging effect on superoxide radical and much weaker effect on hydroxyl free radical, while lipid peroxide in the rat liver microsome was significantly inhibited. In two subsequent studies the fractions with sulfate contents 17.4% and 20.5% were evaluated in vivo in aging mice [48,49]. In both cases, intraperitoneal administration significantly decreased lipid peroxidation in a dose-dependent manner, while at the same time increasing total antioxidant capacity and the activity of SOD and GPx in all organs of the aging mice.

Ulvans of diverse sulfation degree and MW (**269**–**271**) have been isolated from the green alga *Ulva pertusa* and/or chemically modified and their antioxidant activity was tested employing OH and O_2_^−^ radical scavenging, reducing power and metal chelating assays [224,225,226]. Specifically, Qi et al. (2005) extracted ulvan (**269**) with 19.5% sulfate content and chemically prepared derivatives of higher sulfate content ranging from 23.5% to 32.8%. Upon evaluation of their O_2_^−^ radical and OH radical scavenging activity, it was observed that the derivatives displayed higher levels of activity, ranging from 91.7% to 95.5% at a concentration as low as 23.0 μg/mL for O_2_^−^ radical scavenging and with IC_50_ values ranging from 0.46 to 1.43 mg/mL for OH radical scavenging [224].

In another study, Qi et al. (2005) initially extracted ulvan (**270**) from *U. pertusa*, and subsequently, three derivatives of different MW were prepared by H*_2_*O_2_ degradation and their antioxidant activities, including OH and O_2_^−^ radical scavenging activity, reducing power and metal chelating ability, were investigated [225]. The MW of the natural and degraded ulvans were calculated at 151.7, 28.2, 58.0, and 64.5, kDa, respectively. All polysaccharides exhibited significant OH and O_2_^−^ radical scavenging capacity at all concentrations tested with similar IC_50_ values at about >1 mg/mL and 22.1 μg/mL, respectively. Among the natural ulvan and the obtained derivatives, the lowest MW one showed the strongest reducing power and metal chelating ability. The results indicated that MW had a significant effect on the antioxidant activity of ulvan, with low MW ulvan exerting the strongest antioxidant activity. In a further study, Qi et al. (2006) prepared derivatives of ulvan (**262**) after acetylation and benzoylation, which exhibited higher levels of antioxidant activity, as determined using in vitro assays, including scavenging activity against superoxide and hydroxyl radicals, reducing power, and chelating ability [226].

## 8. Miscellaneous Compounds

**Figure 23:**
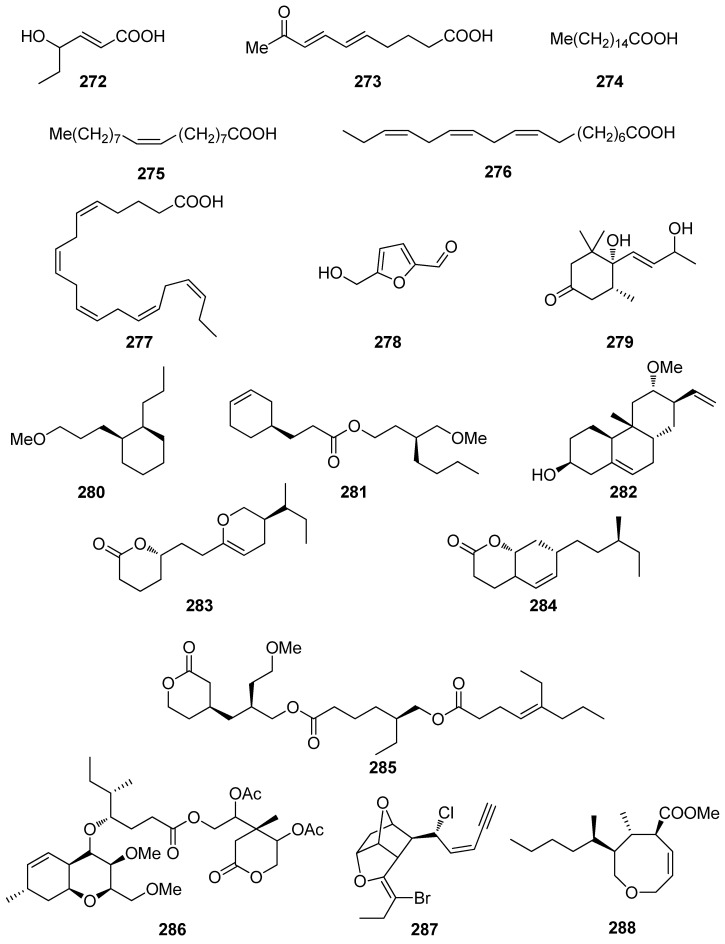
Chemical structures of compounds **272**–**288**.

**Figure 24:**
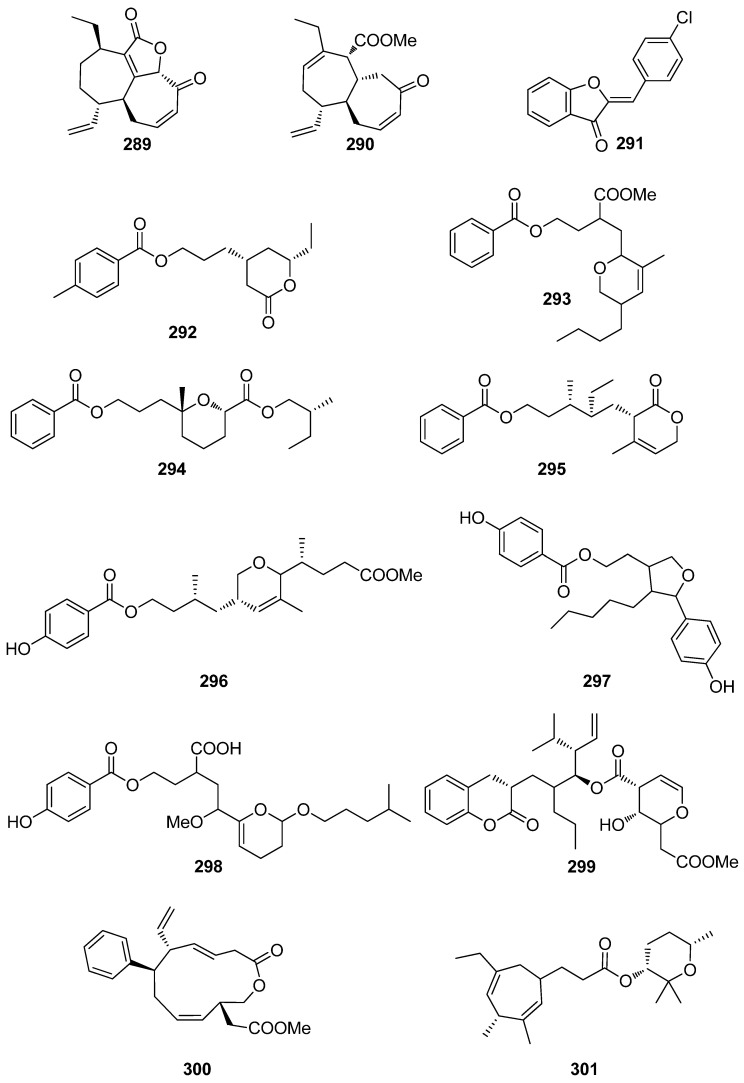
Chemical structures of compounds **289**–**301**.

A number of compounds (**272**–**301**, Figure 23 and Figure 24) isolated from marine macroalgae, displaying various structures that do not belong to the previously described classes, have also exhibited levels of antioxidant activity worth mentioning (Table 7).

Among these, the most active compounds, exerting significant DPPH radical scavenging capacity, were compounds **278** (5-hydroxymethyl-2-furfural, 5-HMF), **291** (Z-4′-chloroaurone), and **297**, with IC_50_ values at 27.1, 22.2, and 25.0 μM, respectively [233,239,241]. In particular, 5-HMF (**278**), isolated from *L. undulata*, exhibited significant antioxidant activities, as evaluated by its in vitro free radical species (including alkyl, DPPH, OH, and O_2_^−^ radicals) scavenging, intracellular ROS scavenging, membrane protein oxidation, MPO inhibition, as well as gene expression of the antioxidative enzymes GSH and SOD [233]. Overall, 5-HMF (**278**) displayed antioxidant activity, by scavenging overproducing free radicals and decreasing the activity of MPO or increasing the activity of GSH and SOD antioxidant enzymes in certain biological pathways.

Fang et al. (2010) isolated the non-polar compounds **272**–**274**, **276**, **277**, and **279** from the red alga *G. furcata* and evaluated their antioxidant activities as inhibitors of AChE and BChE and as scavengers of DPPH radical and ONOO⁻ [75]. All isolated compounds exhibited moderate AChE inhibitory activity with IC_50_ values ranging between 13.6 and 94.4 μM, whereas compounds **276** and **277** showed mild BChE inhibitory activity with IC_50_ values 57.1 and 21.7 μM, respectively. Only compound **272** showed substantial DPPH radical scavenging activity, while compounds **272** and **274** showed potent ONOO⁻ scavenging activity.

Compounds **280**–**286**, **288**–**290**, **292**–**296**, and **298**–**301** exhibited moderate DPPH radical scavenging capacities, with IC_50_ values in the mM range, with activities comparable to either α-tocopherol (IC_50_ = 1.46 mM), or BHT and BHA (IC_50_ ~ 1.30–1.54 mM) [234,235,236,237,238,240,241,242]. Structure–activity relationship analysis revealed that the antioxidant activities of compounds **293**, **297**, and **298** were directly proportional to their steric freedom and hydrophobicity [241].

## 9. Conclusions

The marine environment harbors diverse biological species that can provide a vast repertoire of molecules with therapeutic properties. Forced to tolerate extreme environmental conditions, marine organisms produce structurally unique molecules as an adaptive strategy to survive in their biotopes. In particular, macroalgae contain a plethora of antioxidative compounds, such as bromophenols, phlorotannins, pigments, terpenoids, and polysaccharides, in order to protect themselves from free radicals, the production of which is favored in sublittoral zones with intense exposure to sunlight and high concentrations of oxygen.

Structural elements, such as the number of phenol rings, the number of free hydroxyl groups and conjugated systems, are in general accepted as enhancing the antioxidant activity observed. Among the metabolites presented in the current review, the most active belong to the classes of phenols and polyphenols, as well as meroterpenoids, with bromophenols and phlorotannins exerting the highest activities. In particular, the bromophenol rhodomelin A (**18**) isolated from the red alga *R. confervoides*, the phlorotannins fucodiphloroethol G (**77**), phlorofucofuroeckol-A (**79**), 974-B (**83**), and 2,7″-phloroglucinol-6,6′-bieckol (**84**) purified from brown seaweeds especially of the genus *Ecklonia*, as well as the meroterpenoids **174**, **175**, and **178**–**183** isolated from brown algae of the genus *Sargassum* exerted noticeably high DPPH scavenging activity.

Nevertheless, the most studied antioxidant compounds are the natural pigments astaxanthin (**116**) and fucoxanthin (**118**), belonging to the class of carotenoids, ubiquitous in marine macroalgae. Their antioxidant action is based on their singlet oxygen quenching properties and their free radicals scavenging ability, which mainly depends on the number of conjugated double bonds and end groups. The antioxidant activity of fucoxanthin (**118**) has also been evaluated in vivo. Dietary intake of fucoxanthin significantly reduced lipid hydroperoxide levels of liver and abdominal white adipose tissue of obese/diabetes KK-*A^y^* mice [243]. Fucoxanthin supplementation also significantly reduced the blood glucose level and hepatic lipid contents of the mice. Promising results were also observed in experiments on rats fed a high fat diet supplemented with fucoxanthin that improved the antioxidant capacity, depleted by a high fat diet, by activating the Nrf2 pathway and its downstream target gene NQO1 [244]. Therefore, supplementation of the diet with fucoxanthin, especially of those who consume high fat in their diet, may benefit them by reducing the risk of oxidative stress.

Although emerging evidence points to a diversity of actions and effects, which are intricate and independent from any antioxidant chemical nature, there is an urgent need for deciphering the role of chemical structure on the antioxidant behavior of molecules. Moreover, constraints imposed by experimental protocols should always be taken into consideration when dealing with a lack of biological context in regard to results, so as to discriminate between the in vitro and in vivo scenarios. In this regard, the development of novel antioxidant activity detecting protocols prompts further investigations.

## Figures and Tables

**Table 1:** A list of the most commonly used in vitro assays for the determination of antioxidant activity (adapted from [50]).

**Hydrogen atom transfer (HAT)-based assays**	2,2-azino-bis (3-ethyl benzothiazoline-6-sulfonic acid) diammonium salt (ABTS^+^) radical scavenging [51] β-carotene bleaching [52] crocin bleaching [53] hydrogen peroxide (H_2_O_2_) scavenging [54] hydroxyl radical averting capacity (HORAC) [55] hydroxyl scavenging [56] inhibited oxygen uptake (IOU) [57] lipid peroxidation inhibition capacity (LPIC) [58] oxygen radical absorbance capacity (ORAC) [40] photochemiluminescence (PCL) [59] total radical trapping antioxidant parameter (TRAP) [41]
**Electron transfer (ET)-based assays**	1,1-diphenyl-2-picrylhydrazyl (DPPH) free radical scavenging [46,47] cupric reducing antioxidant capacity (CUPRAC) [60] ferric reducing antioxidant power (FRAP) [44,45] ferric thiocyanate (FTC) [61] nitric oxide radical scavenging [62] *N*,*N*-dimethyl-*p*-phenylene diamine (DMPD) radical scavenging [63] peroxyl radical scavenging [64] potassium ferricyanide reducing power (PFRAP) [65] superoxide anion radical scavenging [66] thiobarbituric acid reactive substances (TBARS) [67] total phenolics content (TPC) using Folin-Ciocalteu reagent [42] trolox equivalence antioxidant capacity (TEAC) using ABTS [43]
**Other in vitro methods**	ascorbic acid content [68] cellular antioxidant activity (CAA) [69] metal chelating activity [70] scavenging of phosphomolybdenum [71] scavenging of xanthine oxidase [72]

**Table 2:** Phenolic compounds from macroalgae with antioxidant activity.

Compound	Isolation Source	Assay/Activity	Reference
**1**	*Symphyocladia latiuscula* (Rhodophyta, Florideophyceae, Ceramiales)	DPPH scavenging: IC_50_ = 14.0 μM	[74]
**2**	*Gloiopeltis furcata* (Rhodophyta, Florideophyceae, Gigartinales)	DPPH scavenging: IC_50_ = 86.2 μΜ ONOO^−^ scavenging: 4.58 ± 0.01 μM	[75]
**3**	*Rhodomela confervoides* (Rhodophyta, Florideophyceae, Ceramiales)	ABTS^+^ scavenging: IC_50_ = 1.60 ± 0.04 μM DPPH scavenging: IC_50_ = 50.6 ± 0.2 μM	[76]
**4**	*R. confervoides* (Rhodophyta, Florideophyceae, Ceramiales)	ABTS^+^ scavenging: IC_50_ = 1.56 ± 0.02 μM DPPH scavenging: IC_50_ = 42.3 ± 0.2 μM; 67%	[76,77]
**5**	*S. latiuscula* (Rhodophyta, Florideophyceae, Ceramiales)	bleomycin-dependent DNA damage deoxyribose assay	[78]
**6**	*R. confervoides* (Rhodophyta, Florideophyceae, Ceramiales)	ABTS^+^ scavenging: IC_50_ = 1.62 ± 0.03 μM DPPH scavenging: IC_50_ = 40.5 ± 0.2 μM; 30%	[76,77]
**7**	*S. latiuscula* (Rhodophyta, Florideophyceae, Ceramiales)	DPPH scavenging: IC_50_ = 15.5 μM	[74]
**8**	*S. latiuscula* (Rhodophyta, Florideophyceae, Ceramiales)	DPPH scavenging: IC_50_ = 7.5 µM	[79]
**9**	*R. confervoides* (Rhodophyta, Florideophyceae, Ceramiales)	ABTS^+^ scavenging: IC_50_ = 1.36 ± 0.01 μM DPPH scavenging: IC_50_ = 38.4 ± 0.2 μM	[76]
**10**	*R. confervoides* (Rhodophyta, Florideophyceae, Ceramiales)	ABTS^+^ scavenging: IC_50_ = 2.11 ± 0.04 μM DPPH scavenging: IC_50_ = 7.43 ± 0.10 μM	[76]
**11**	*R. confervoides* (Rhodophyta, Florideophyceae, Ceramiales)	ABTS^+^ scavenging: IC_50_ = 1.87 ± 0.02 μM DPPH scavenging: IC_50_ = 20.5 ± 0.1 μM	[76]
**12**	*S. latiuscula* (Rhodophyta, Florideophyceae, Ceramiales)	ABTS^+^ scavenging: IC_50_ = 71.0 μM DPPH scavenging: IC_50_ = 14.4; 18.5 μM CUPRAC Fe^2+^ chelation: IC_50_ = 44.7 μM FRAP AChE inhibition: IC_50_ = 13.85 nM BChE inhibition: IC_50_ = 38.22 nM	[74,80]
**13**	*R. confervoides* (Rhodophyta, Florideophyceae, Ceramiales)	ABTS^+^ scavenging: IC_50_ = 8.07 μM; TEAC = 2.68 mM DPPH scavenging: IC_50_ = 12.4; 15.9 μM CUPRAC Fe^2+^ chelation: IC_50_ = 65.2 μM FRAP AChE inhibition: IC_50_ = 17.10 nM BChE inhibition: IC_50_ = 40.57 nM	[80,81]
**14**	*R. confervoides* (Rhodophyta, Florideophyceae, Ceramiales)	ABTS^+^ scavenging: IC_50_ = 8.1 μM; TEAC = 2.21 mM DPPH scavenging: IC_50_ = 14.6; 18.5 μM CUPRAC Fe^2+^ chelation: IC_50_ = 54.6 μM FRAP AChE inhibition: IC_50_ = 29.88 nM BChE inhibition: IC_50_ = 46.51 nM	[80,81]
**15**	*R. confervoides* (Rhodophyta, Florideophyceae, Ceramiales)	ABTS^+^ scavenging: TEAC = 2.31 mM DPPH scavenging: IC_50_ = 5.43 μM	[81]
**16**	*R. confervoides* (Rhodophyta, Florideophyceae, Ceramiales)	ABTS^+^ scavenging: TEAC = 2.14 mM DPPH scavenging: IC_50_ = 5.70 μM	[81]
**17**	*S. latiuscula* (Rhodophyta, Florideophyceae, Ceramiales)	DPPH scavenging: IC_50_ = 27.9 μM	[82]
**18**	*R. confervoides* (Rhodophyta, Florideophyceae, Ceramiales)	ABTS^+^ scavenging: TEAC = 4.37 ± 0.24 mM DPPH scavenging: IC_50_ = 3.82 ± 0.01 μM	[83]
**19**	*R. confervoides* (Rhodophyta, Florideophyceae, Ceramiales)	ABTS^+^ scavenging: TEAC = 2.06 ± 0.08 mM DPPH scavenging: IC_50_ = 9.52 ± 0.04 μM	[76]
**20**	*S. latiuscula* (Rhodophyta, Florideophyceae, Ceramiales)	DPPH scavenging: IC_50_ = 24.0 μM	[74]
**21**	*Polysiphonia morrowii*, *Polysiphonia urceolata*, *R. confervoides* (Rhodophyta, Florideophyceae, Ceramiales)	DPPH scavenging: IC_50_ = 20.3 μM cytoprotective effect against cellular oxidative stress HO-1 activity and expression in keratinocytes Nrf2 expression Nrf2 nuclear translocation	[84,85]
**22**	*R. confervoides* (Rhodophyta, Florideophyceae, Ceramiales)	ABTS^+^ scavenging: TEAC = 1.32 ± 0.02 mM DPPH scavenging: IC_50_ = 58.2 ± 0.4 μM	[76]
**23**	*P. urceolata* (Rhodophyta, Florideophyceae, Ceramiales)	DPPH scavenging: IC_50_ = 35.8 μM	[84]
**24**	*R. confervoides*, *Vertebrata lanosa* (Rhodophyta, Florideophyceae, Ceramiales)	ABTS^+^ scavenging: TEAC = 1.09 ± 0.01 mM CAA CLPAA DPPH scavenging: IC_50_ = 32.0 ± 0.1 μM ORAC	[76,86]
**25**	*S. latiuscula* (Rhodophyta, Florideophyceae, Ceramiales)	DPPH scavenging: IC_50_ = 24.7 μM	[74]
**26**	*Cladophora wrightiana* (Chlorophyta, Ulvophyceae, Cladophorales)	DPPH scavenging: 69% at 160 μM OH scavenging O_2_^−^ scavenging protective effect against UVB-induced apoptosis and DNA damage in HaCaT cells scavenging activity against H_2_O_2_- or UVB-generated intracellular ROS in HaCaT cells	[87]
**27**	*R. confervoides* (Rhodophyta, Florideophyceae, Ceramiales)	ABTS^+^ scavenging: TEAC = 1.86 ± 0.02 mM DPPH scavenging: IC_50_ = 50.3 ± 0.3 μM	[76]
**28**	*R. confervoides* (Rhodophyta, Florideophyceae, Ceramiales)	ABTS^+^ scavenging: TEAC = 2.11 mM DPPH scavenging: IC_50_ = 23.6 μM	[81]
**29**	*R. confervoides* (Rhodophyta, Florideophyceae, Ceramiales)	ABTS^+^ scavenging: TEAC = 1.98 ± 0.01 mM DPPH scavenging: IC_50_ = 30.9 ± 0.1 μM	[76]
**30**	*R. confervoides* (Rhodophyta, Florideophyceae, Ceramiales)	ABTS^+^ scavenging: TEAC = 2.35 ± 0.02 mM DPPH scavenging: IC_50_ = 26.3 ± 0.2 μM	[76]
**31**	*R. confervoides* (Rhodophyta, Florideophyceae, Ceramiales)	ABTS^+^ scavenging: TEAC = 2.87 ± 0.11 mM DPPH scavenging: IC_50_ = 19.8 ± 0.1 μM	[76]
**32**	*R. confervoides* (Rhodophyta, Florideophyceae, Ceramiales)	ABTS^+^ scavenging: TEAC = 2.07 ± 0.12 mM DPPH scavenging: IC_50_ = 30.2 ± 0.2 μM	[76]
**33**	*P. urceolata* (Rhodophyta, Florideophyceae, Ceramiales)	DPPH scavenging: IC_50_ = 16.1 ± 0.1 μM	[88]
**34**	*R. confervoides* (Rhodophyta, Florideophyceae, Ceramiales)	ABTS^+^ scavenging: TEAC = 2.36 mM DPPH scavenging: IC_50_ = 20.8 μM	[81]
**35**	*R. confervoides* (Rhodophyta, Florideophyceae, Ceramiales)	ABTS^+^ scavenging: TEAC = 2.11 ± 0.11 mM DPPH scavenging: IC_50_ = 18.6 ± 0.1 μM	[76]
**36**	*R. confervoides* (Rhodophyta, Florideophyceae, Ceramiales)	ABTS^+^ scavenging: TEAC = 1.63 ± 0.01 mM DPPH scavenging: IC_50_ = 50.9 ± 0.3 μM	[76]
**37**	*R. confervoides* (Rhodophyta, Florideophyceae, Ceramiales)	ABTS^+^ scavenging: TEAC = 3.68 ± 0.12 mM DPPH scavenging: IC_50_ = 8.72 ± 0.05 μM	[76]
**38**	*P. urceolata*, *R. confervoides* (Rhodophyta, Florideophyceae, Ceramiales)	ABTS^+^ scavenging: TEAC = 3.10 ± 0.13 mM DPPH scavenging: IC_50_ = 9.40 ± 0.05; 9.67 ± 0.04 μM	[76,88]
**39**	*R. confervoides* (Rhodophyta, Florideophyceae, Ceramiales)	ABTS^+^ scavenging: TEAC = 3.45 ± 0.12 mM DPPH scavenging: IC_50_ = 7.62 ± 0.01 μM	[76]
**40**	*S. latiuscula* (Rhodophyta, Florideophyceae, Ceramiales)	DPPH scavenging: IC_50_ = 43.8 μM	[82]
**41**	*S. latiuscula* (Rhodophyta, Florideophyceae, Ceramiales)	DPPH scavenging: IC_50_ = 8.5 µM	[79]
**42**	*R. confervoides* (Rhodophyta, Florideophyceae, Ceramiales)	ABTS^+^ scavenging: TEAC = 2.87 mM DPPH scavenging: IC_50_ = 5.22 μM	[81]
**43**	*Odonthalia corymbifera* (Rhodophyta, Florideophyceae, Ceramiales)	ABTS^+^ scavenging: IC_50_ = 17.3 ± 0.1 μM Cu^2+^-chelation: IC_50_ = 61.9 ± 0.1 μM CUPRAC: EC_A0.50_ = 13.6 ± 0.1 μM DPPH scavenging: IC_50_ = 24.7 ± 0.0 μM FRAP: EC_A0.50_ = 11.1 ± 0.1 μM tyrosinase inhibition: IC_50_ = 17.3 ± 0.1 μM	[89]
**44**	*P. morrowii* (Rhodophyta, Florideophyceae, Ceramiales)	LPS-induced ROS generation and ROS-mediated ERK signaling in RAW 264.7 macrophages	[90]
**45**	*R. confervoides*, *V. lanosa* (Rhodophyta, Florideophyceae, Ceramiales)	ABTS^+^ scavenging: TEAC = 3.05 mM CAA CLPAA DPPH scavenging: IC_50_ = 17.6 μM ORAC	[81,86]
**46**	*S. latiuscula* (Rhodophyta, Florideophyceae, Ceramiales)	DPPH scavenging: IC_50_ = 8.5 μM	[74]
**47**	*R. confervoides* (Rhodophyta, Florideophyceae, Ceramiales)	ABTS^+^ scavenging: TEAC = 3.18 mM DPPH scavenging: IC_50_ = 16.9 μM; 27%	[77,81]
**48**	*S. latiuscula* (Rhodophyta, Florideophyceae, Ceramiales)	DPPH scavenging: IC_50_ = 8.1 μM	[74]
**49**	*Avrainvillea* sp. (Chlorophyta, Ulvophyceae, Bryopsidales)	DPPH scavenging: strong exogenous ROS scavenging in TPA-treated HL-60 cells (DCFH-DA): IC_50_ = 6.1 μM	[91]
**50**	*R. confervoides*, *V. lanosa* (Rhodophyta, Florideophyceae, Ceramiales)	ABTS^+^ scavenging: TEAC = 3.16 mM CAA CLPAA DPPH scavenging: IC_50_ = 19.6 μM ORAC	[81,86]
**51**	*R. confervoides* (Rhodophyta, Florideophyceae, Ceramiales)	ABTS^+^ scavenging: TEAC = 3.00 mM DPPH scavenging: IC_50_ = 14.3 μM	[81]
**52**	*R. confervoides* (Rhodophyta, Florideophyceae, Ceramiales)	ABTS^+^ scavenging: TEAC = 2.78 mM DPPH scavenging: IC_50_ = 13.8 μM	[81]
**53**	*S. latiuscula* (Rhodophyta, Florideophyceae, Ceramiales)	DPPH scavenging: ΙC_50_ = 10.5 μM	[74]
**54**	*O. corymbifera* (Rhodophyta, Florideophyceae, Ceramiales)	ABTS^+^ scavenging: IC_50_ = 6.7 ± 0.1 μM Cu^2+^-chelation: IC_50_ = 74.3 ± 0.1 μM CUPRAC: EC_A0.50_ = 7.8 ± 0.1 μM DPPH scavenging: IC_50_ = 13.5 ± 0.0 μM FRAP: EC_A0.50_ = 10.8 ± 0.1 μM tyrosinase inhibition: ΙC_50_ = 31.0 ± 0.1 μM	[90]
**55**	*V. lanosa* (Rhodophyta, Florideophyceae, Ceramiales)	CAA CLPAA ORAC	[86]
**56**	*R. confervoides* (Rhodophyta, Florideophyceae, Ceramiales)	ABTS^+^ scavenging: TEAC = 3.21 mM DPPH scavenging: IC_50_ = 13.6 μM	[81]
**57**	*P. urceolata* (Rhodophyta, Florideophyceae, Ceramiales)	DPPH scavenging: IC_50_ = 19.6 ± 0.1 μM	[88]
**58**	*P. urceolata* (Rhodophyta, Florideophyceae, Ceramiales)	DPPH scavenging: IC_50_ = 21.9 ± 0.1 μM	[88]
**59**	*S. latiuscula* (Rhodophyta, Florideophyceae, Ceramiales)	DPPH scavenging: IC_50_ = 10.2 μM	[74]
**60**	*P. urceolata* (Rhodophyta, Florideophyceae, Ceramiales)	DPPH scavenging: IC_50_ = 8.1 μM	[84]
**61**	*P. urceolata* (Rhodophyta, Florideophyceae, Ceramiales)	DPPH scavenging: IC_50_ = 15.1 μM	[84]
**62**	*P. urceolata* (Rhodophyta, Florideophyceae, Ceramiales)	DPPH scavenging: IC_50_ = 6.8 μM	[84]
**63**	*P. urceolata* (Rhodophyta, Florideophyceae, Ceramiales)	DPPH scavenging: IC_50_ = 6.1 μM	[84]
**64**	*P. urceolata* (Rhodophyta, Florideophyceae, Ceramiales)	DPPH scavenging: IC_50_ = 7.9 μM	[92]
**65**	*R. confervoides* (Rhodophyta, Florideophyceae, Ceramiales)	ABTS^+^ scavenging: TEAC = 3.58 mM DPPH scavenging: IC_50_ = 8.90 μM	[81]
**66**	*Sargassum wightii*, *Sargassum tenerrimum*, *Turbinaria conoides* (Ochrophyta, Phaeophyceae, Fucales) *Ishige okamurae* (Ochrophyta, Phaeophyceae, Ishigeales) *Ecklonia cava* (Ochrophyta, Phaeophyceae, Laminariales)	alkyl scavenging: IC_50_ = 103.5 ± 1.9 μM DPPH scavenging: 64.71–71.07% at 200 μg/mL H_2_O_2_ scavenging: 88.33–89.7% at 200 μg/mL OH scavenging: IC_50_ = 392.5 ± 2.8; 408.5 ± 3.7 μM O_2_^−^ scavenging: IC_50_ = 115.2 ± 2.5; 124.7 ± 2.4 μM ROO scavenging: IC_50_ = 128.9 ± 2.2 μM metal chelating activity: 11.40–14.38% at 200 μg/mL H_2_O_2_-induced apoptosis, cytotoxicity, DNA damage, mitochondrial dysfunction and ROS generation in HaCaT keratinocytes intracellular ROS generation (DCFH-DA) in RAW 264.7 macrophages/V79-4 cells Nrf2/HO-1 signaling pathway in HaCaT keratinocytes	[93,94,95,96,97]
**67**	*Gracilaria* sp. (Rhodophyta, Florideophyceae, Gracilariales)	DPPH scavenging: 83.8 ± 2.6% XO inhibition: 64.7 ± 0.7%	[98]
**68**	*Sargassum micracanthum* (Ochrophyta, Phaeophyceae, Fucales)	ABTS^+^ scavenging: IC_50_ = 47 μM	[99]
**69**	*E. cava* (Ochrophyta, Phaeophyceae, Laminariales)	oxidative stress-induced DNA damage in V79-4 cells	[100]
**70**	*Ishige foliacea* (Ochrophyta, Phaeophyceae, Ishigeales)	enzyme activity (SOD, CAT, GPx) intracellular ROS generation and lipid peroxidation in HUVEC/pancreatic β cells oxidative stress-induced cell death in zebrafish embryo streptozotocin-induced pancreatic β cell damage in rat insulinoma cell line	[101,102]
**71**	*E. cava*, *Ecklonia kurome*, *Ecklonia stolonifera*, *Eisenia bicyclis* (Ochrophyta, Phaeophyceae, Laminariales)	DPPH scavenging: IC_50_ = 11.5; 22.9 ± 0.52; 26 µM OH scavenging: IC_50_ = 51.8 ± 2.5 µM O_2_^−^ scavenging: IC_50_ = 26.5 ± 1.25; 107 µM ROO scavenging: IC_50_ = 28.4 ± 1.5 µM inhibitory effect on total ROS: IC_50_ = 4.04 ± 0.04 µM cellular membrane protein oxidation in RAW 264.7 macrophages GSH levels in HepG2 cells/RAW 264.7 macrophages HO-1 expression H_2_O_2_-induced lipid peroxidation (TBARS) in V79-4 cells intracellular ROS generation (DCFH-DA) and oxidative stress induced cell damage in lung fibroblast cells MPO activity in HL60 cells Nrf2 nuclear translocation and activation PM_10_ (particulate matter of less than 10 mm) -induced lipid peroxidation and cytokine expression in human epidermal keratinocytes	[95,103,104,105,106,107,108]
**72**	*E. stolonifera* (Ochrophyta, Phaeophyceae, Laminariales)	DPPH scavenging: IC_50_ = 8.8 ± 0.4 μM intracellular ROS scavenging	[109]
**73**	*I. okamurae* (Ochrophyta, Phaeophyceae, Ishigeales)	alkyl scavenging: IC_50_ = 18.8 ± 1.2 μM DPPH scavenging: IC_50_ = 10.5 ± 0.5 μM OH scavenging: IC_50_ = 27.1 ± 0.9 μM O_2_^−^ scavenging: IC_50_ = 16.7 ± 0.6 μM H_2_O_2_-induced oxidative stress-induced ROS generation (DCFH-DA) in murine hippocampal neuronal cells intracellular Ca^2+^ level lipid peroxidation assay (TBARS) membrane protein oxidation MPO activity PM_2.5_ (fine particulate matter with a diameter ≤2.5 μm) -induced ROS generation in human keratinocytes PM_2.5_-induced DNA damage, endoplasmic reticulum stress and autophagy, mitochondrial damage, apoptosis via MAPK signaling pathways	[97,110,111]
**74**	*E. cava* (Ochrophyta, Phaeophyceae, Laminariales)	DPPH scavenging: IC_50_ = 18.6 ± 1.0 μM OH scavenging: IC_50_ = 39.6± 2.1 μM O_2_^−^ scavenging: IC_50_ = 21.9 ± 1.8 μM ROO scavenging: IC_50_ = 22.7 ± 1.5 μM cellular membrane protein oxidation in RAW 264.7 cells GSH levels in RAW 264.7 cells intracellular ROS generation (DCFH-DA) MPO activity in HL60 cells	[95]
**75**	*E. cava*, *E. kurome*, *E. stolonifera*, *E. bicyclis* (Ochrophyta, Phaeophyceae, Laminariales)	DPPH scavenging: IC_50_ = 6.2 ± 0.4; 8.28 ± 0.45; 13 μM OH scavenging: IC_50_ = 28.6 ±2.5 μM O_2_^−^ scavenging: IC_50_ = 7.6; 16.2 ±1.0 μM ROO scavenging: IC_50_ = 14.5 ±1.8 μM apoptosis in Hep3B cells cellular membrane protein oxidation in RAW 264.7 cells detection of apoptosis-related proteins GSH levels in RAW 264.7 cells intracellular ROS generation (DCFH-DA) in RAW 264.7 cells MPO activity in HL60 cells PM_10_ (particulate matter of less than 10 mm) -induced lipid peroxidation and cytokine expression in human epidermal keratinocytes rotenone-induced oxidative stress in SH-SY5Y cells	[95,107,108,109,112,113]
**76**	*Fucus spiralis* (Ochrophyta, Phaeophyceae, Fucales)	DPPH scavenging: Q_50_ = 0.090 ± 0.002 μmol	[114]
**77**	*E. cava* (Ochrophyta, Phaeophyceae, Laminariales)	DPPH scavenging: IC_50_ = 0.60; 14.7 ± 1.2 μM OH scavenging: IC_50_ = 3.5 ± 1.55 μM O_2_^−^ scavenging: IC_50_ = 18.6 ± 1.5 μM ROO scavenging: IC_50_ = 18.1 ± 1.0 μM cellular membrane protein oxidation in RAW 264.7 cells GSH levels in RAW 264.7 cells intracellular ROS generation (DCFH-DA) intracellular ROS detection in UVB-irradiated HaCaT keratinocytes MPO activity in HL60 cells	[95,115,116]
**78**	*Fucus vesiculosus* (Ochrophyta, Phaeophyceae, Fucales)	DPPH scavenging: ΙC_50_ = 16.1 ± 1.0 μM O_2_^−^ scavenging: ΙC_50_ > 401.6 μM ORAC: 3.3 ± 0.3 units at 1 μg/mL	[117]
**79**	*E. cava*, *E. kurome*, *E. stolonifera*, *E. bicyclis* (Ochrophyta, Phaeophyceae, Laminariales)	alkyl scavenging: IC_50_ = 3.9 μM DPPH scavenging: IC_50_ = 4.7 ± 0.3; 10.3; 12; 17.7 ± 0.8 μM OH scavenging: IC_50_ = 21.4; 39.2± 1.8 μM O_2_^−^ scavenging: IC_50_ = 8.4 μM; IC_50_ = 21.6 ± 2.2 μM ROO scavenging: IC_50_ = 21.4 ± 2.1 μM total ROS generation: IC_50_ = 3.80 ± 0.09 μM intracellular ROS generation (DCFH-DA) in RAW 264.7 macrophages/Vero cells/zebrafish system	[95,105,108,109,118]
**80**	*I. okamurae* (Ochrophyta, Phaeophyceae, Ishigeales) *E. cava*, *E. bicyclis* (Ochrophyta, Phaeophyceae, Laminariales) *Grateloupia elliptica* (Rhodophyta, Florideophyceae, Halymeniales)	ABTS^+^ scavenging: IC_50_ = 37.1 ± 2.8 μΜ alkyl scavenging: IC_50_ = 17.3 ± 1.0 μM DPPH scavenging: IC_50_ = 8.69 ± 0.35; 9.1 ± 0.4; 28; 66.5 ± 0.5 μΜ OH scavenging: IC_50_ = 28.7 ± 1.1; 29.7 ± 1.5 μM O_2_^−^ scavenging: IC_50_ = 15.4 ± 0.9; 15.9 ± 1.3 μM ROO scavenging: IC_50_ = 17.1 ± 2.2 μM singlet oxygen (^1^O_2_) quenching: QC_50_ = 30.7 ± 2.4 μM cellular membrane protein oxidation in RAW 264.7 macrophages GSH levels in RAW 264.7 macrophages high-glucose-induced oxidative stress intracellular ROS generation (DCFH-DA) in UVB-irradiated HaCaT keratinocytes MPO activity in HL60 cells	[95,97,119,120,121]
**81**	*E. bicyclis* (Ochrophyta, Phaeophyceae, Laminariales)	ABTS^+^ scavenging: IC_50_ = 43.3 ± 2.3 μΜ DPPH scavenging: IC_50_ = 103.0 ± 3.5 μM singlet oxygen (^1^O_2_) quenching: QC_50_ = 35.7 ± 2.4 μM	[119]
**82**	*E. cava*, *E. kurome*, *E. bicyclis* (Ochrophyta, Phaeophyceae, Laminariales)	ABTS^+^ scavenging: IC_50_ = 43.4 ± 2.0 μΜ DPPH scavenging: IC_50_ = 15.0; 95.9 ± 3.2 μΜ O_2_^−^ scavenging: IC_50_ = 6.5 μM singlet oxygen (^1^O_2_) quenching: QC_50_ = 49.4 ± 1.7 μM H_2_O_2_-induced DNA damage intracellular ROS generation in Vero cells	[108,119]
**83**	*E. bicyclis* (Ochrophyta, Phaeophyceae, Laminariales)	DPPH scavenging: IC_50_ = 0.86 ± 0.02 μM ONOO^−^ scavenging: 1.80 ± 0.01 μM total ROS: 6.45 ± 0.04 μM	[122]
**84**	*E. cava* (Ochrophyta, Phaeophyceae, Laminariales)	alkyl scavenging: IC_50_ = 2.07 ± 1.00 μM DPPH scavenging: IC_50_ = 0.51 μM OH scavenging: IC_50_ = 75.6μM O_2_^−^ scavenging: IC_50_ = 57.2 μM intracellular ROS generation (DCFH-DA) in H_2_O_2_-treated Vero cells	[123]
**85**	*F. spiralis* (Ochrophyta, Phaeophyceae, Fucales)	DPPH scavenging: Q_50_ = 0.087 ± 0.004 μmol	[114]
**86**	*F. vesiculosus* (Ochrophyta, Phaeophyceae, Fucales)	DPPH scavenging: ΙC_50_ = 19.3 ± 2.7 μM O_2_^−^ scavenging: ΙC_50_ > 334.9 μM ORAC: 3.5 ± 0.2 units at 1 μg/mL	[117]
**87**	*F. vesiculosus* (Ochrophyta, Phaeophyceae, Fucales)	DPPH scavenging: IC_50_ = 15.8 ± 1.5 μM O_2_^−^ scavenging: IC_50_ > 175.6 μM ORAC: 3.2 ± 0.2 units at 1 μg/mL	[117]
**88**	*Acanthophora spicifera* (Rhodophyta, Florideophyceae, Ceramiales)	lipid peroxidation and inhibition of the generation of MDA in rat liver: IC_50_ = 1.0 × 10^−2^ μM	[124]
**89**	*A. spicifera* (Rhodophyta, Florideophyceae, Ceramiales)	lipid peroxidation and inhibition of the generation of MDA in rat liver: IC_50_ = 1.5 × 10^−2^ μM	[124]

ABTS^+^: 2,2’-azino-bis (3-ethyl benzothiazoline-6-sulfonic acid) diammonium salt; AChE: acetylcholinesterase; BChE: butyrylcholinesterase; CAA: cellular antioxidant activity; CAT: catalase; CLPAA: cellular lipid peroxidation antioxidant activity; CUPRAC: cupric reducing antioxidant capacity; DCFH-DA: cell-based 2′,7′-dichlorodihydrofluorescein diacetate antioxidant assay; DPPH: 1,1-diphenyl-2-picrylhydrazyl free radical; EC_A0.50_: effective concentration for absorbance of 0.50; FRAP: ferric reducing antioxidant power; GSH: glutathione; GPx: glutathione peroxidase; HO-1: heme oxygenase-1; H_2_O_2_: hydrogen peroxide; IC_50_: half maximal inhibitory concentration; LPS: lipopolysaccharide; MAPK: mitogen-activated protein kinase; MDA: malondialdehyde; MPO: myeloperoxidase; Nrf2: nuclear factor erythroid 2-related factor 2; OH: hydroxyl; ONOO^−^: peroxynitrite; O_2_^−^: superoxide anion; ORAC: oxygen radical absorbance capacity; Q_50_: amount of phenolics (in μg) necessary to obtain 50% of inhibition in the DPPH assay; QC_50_: half maximal quenching concentration; ROO: peroxyl; ROS: reactive oxygen species; SH-SY5Y: human dopaminergic neuronal cell line; SOD: superoxide dismutase; TBARS: thiobarbituric acid reactive substances; TEAC: trolox equivalence antioxidant capacity; TPA: 12-*O*-tetradecanoylphorbol 13-acetate; V79-4: Chinese hamster lung fibroblast cell line; XO: xanthine oxidase.

**Table 3:** Terpenoids from macroalgae with antioxidant activity.

Compound	Isolation Source	Assay/Activity	Reference
**90**	*Plocamium* sp. (Rhodophyta, Florideophyceae, Plocamiales)	DPPH scavenging: IC_50_ = 0.05 ± 0.01 mM H_2_O_2_ scavenging: IC_50_ = 5.58 ± 1.11 mM NO scavenging: IC_50_ = 4.18 ± 0.22 mM reducing power (Fe^3+^ to Fe^2+^ reduction)	[128]
**91**	*Ulva fasciata* (Chlorophyta, Ulvophyceae, Ulvales)	ABTS^+^ scavenging: 66.8 ± 1.5% at 50 μM DPPH scavenging: IC_50_ = 13.74 ± 1.38 mM	[129]
**92**	*Pyropia orbicularis* (Rhodophyta, Bangiophyceae, Bangiales)	activation of antioxidant responses during desiccation	[130]
**93**	*U. fasciata* (Chlorophyta, Ulvophyceae, Ulvales)	ABTS^+^ scavenging DPPH scavenging: IC_50_ = 80.56 ± 2.43 mM	[129]
**94**	*U. fasciata* (Chlorophyta, Ulvophyceae, Ulvales)	ABTS^+^ scavenging DPPH scavenging: IC_50_ = 23.60 ± 1.15 mM	[129]
**95**	*U. fasciata* (Chlorophyta, Ulvophyceae, Ulvales)	ABTS^+^ scavenging DPPH scavenging: IC_50_ = 20.83 ± 0.92 mM	[129]
**96**	*U. fasciata* (Chlorophyta, Ulvophyceae, Ulvales)	ABTS^+^ scavenging: 78 ± 1.9% at 50 μM DPPH scavenging: IC_50_ = 10.24 ± 0.98 mM	[129]
**97**	*Laurencia tristicha* (Rhodophyta, Florideophyceae, Ceramiales)	alcohol-induced oxidative injury in rats enzyme activity (SOD, CAT, GPx) D-galactose-induced oxidation in mice endogenous apoptosis-related genes’ expression (BAX, cytochrome c, cytochrome P450, BCL-2, Caspase-9 and Caspase-3) GSH content lipid peroxidation	[131,132]
**98**	*Laurencia dendroidea* (Rhodophyta, Florideophyceae, Ceramiales)	DPPH scavenging: 30.3% at 2.12 mM H_2_O_2_ scavenging	[133]
**99**	*L. dendroidea* (Rhodophyta, Florideophyceae, Ceramiales)	DPPH scavenging: 27.5% at 2.12 mM H_2_O_2_ scavenging	[133]
**100**	*S. wightii* (Ochrophyta, Phaeophyceae, Fucales)	ABTS^+^ scavenging IC_50_ = 1.18 ± 0.07 mM DPPH scavenging: IC_50_ = 1.08 ± 0.07 mM	[134]
**101**	*S. wightii* (Ochrophyta, Phaeophyceae, Fucales)	ABTS^+^ scavenging: IC_50_ = 0.72 ± 0.09 mM DPPH scavenging: IC_50_ = 0.75 ± 0.03 mM	[134]
**102**	*Cystoseira trinodis* (Ochrophyta, Phaeophyceae, Fucales)	ABTS^+^ scavenging: 24.19 ± 1.15% inhibition at 2 mM	[135]
**103**	*C. trinodis* (Ochrophyta, Phaeophyceae, Fucales)	ABTS^+^ scavenging: 27.50 ± 1.30% inhibition at 2 mM	[135]
**104**	*C. trinodis* (Ochrophyta, Phaeophyceae, Fucales) *E. stolonifera*, *E. bicyclis* (Ochrophyta, Phaeophyceae, Laminariales)	ABTS^+^ scavenging: 24.05 ± 2.38% inhibition at 2 mM intracellular ROS generation (DCFH-DA) intracellular GSH levels in t-BHP- and tacrine-treated HepG2 cells t-BHP- and tacrine-induced oxidative stress in HepG2 cells	[135,136]
**105**	*C. trinodis* (Ochrophyta, Phaeophyceae, Fucales)	ABTS^+^ scavenging: 26.37 ± 0.20% inhibition at 2 mM	[135]
**106**	*C. trinodis* (Ochrophyta, Phaeophyceae, Fucales)	ABTS^+^ scavenging: 20.41 ± 0.13% inhibition at 2 mM	[135]
**107**	*Caulerpa racemosa* (Chlorophyta, Ulvophyceae, Bryopsidales)	Alkyl scavenging: IC_50_ = 0.66 ± 0.05 mM OH scavenging: IC_50_ = 0.29 ± 0.05 mM	[137]
**108**	*S. wightii* (Ochrophyta, Phaeophyceae, Fucales)	ABTS^+^ scavenging IC_50_ = 0.37 ± 0.02 mM DPPH scavenging: IC_50_ = 0.31 ± 0.02 mM	[134]
**109**	*S. wightii* (Ochrophyta, Phaeophyceae, Fucales)	ABTS^+^ scavenging: IC_50_ = 0.37 ± 0.02 mM DPPH scavenging: IC_50_ = 0.34 ± 0.06 mM	[134]
**110**	*Gracilaria salicornia* (Rhodophyta, Florideophyceae, Gracilariales)	ABTS^+^ scavenging: IC_50_ = 1.09 mM DPPH scavenging: IC_50_ = 1.33 mM	[138]
**111**	*G. salicornia* (Rhodophyta, Florideophyceae, Gracilariales)	ABTS^+^ scavenging: IC_50_ = 1.24 mM DPPH scavenging: IC_50_ = 1.56 mM	[138]
**112**	from plants and microalgae, but also from macroalgae	enzyme activity (CAT, SOD, GPx and GSH reductase) GSH and TBARS levels in hepatic tissue of lycopene-treated rats	[139]
**113**	from plants and microalgae, but also from macroalgae	intracellular ROS generation in LPS-stimulated RAW 264.7 macrophages LPS- and IFN-γ-induced NO generation in RAW 264.7 macrophages TPA-induced O_2_^−^ generation in differentiated human promyelocytic HL-60 cells	[140,141,142]
**114**	from plants and microalgae, but also from macroalgae	LPS- and IFN-γ-induced NO generation in RAW 264.7 macrophages TPA-induced O_2_^−^ generation in differentiated human promyelocytic HL-60 cells	[141,142]
**115**	from plants and microalgae, but also from macroalgae	LPS- and IFN-γ-induced NO generation in RAW 264.7 macrophages TPA-induced O_2_^−^ generation in differentiated human promyelocytic HL-60 cells	[141,142]
**116**	from plants and microalgae, but also from macroalgae	radical scavenging enzyme (SOD2, CAT, and GPx1) regulation in irradiated cells intracellular ROS generation (DCFH-DA) in acetaldehyde-treated SH-SY5Y cells LPS- and IFN-γ-induced NO generation in RAW 264.7 macrophages Nrf2/HO-1 antioxidant pathway Nrf2 dissociation and nuclear translocation Nrf2 expression regulation in irradiated cells Nrf2-regulated enzymes expression (HO-1, NQO-1, and GST-α1) PI3K/Akt and ERK signaling pathway regulation ROS-induced oxidative stress in a rat deep-burn model regulation of free radical production (XO/reduced form of Nox) Sp1/NR1 signaling pathway regulation TPA-induced O_2_^−^ generation in differentiated human promyelocytic HL-60 cells Akt/CREB and p38 kinase/MAPK signaling pathway in acetaldehyde-treated SH-SY5Y cells	[141,142,143,144,145,146,147,148,149,150,151,152]
**117**	from plants and microalgae, but also from macroalgae	ROO scavenging (ORAC/ESR) caspase-3/7 activation Nrf2/ARE signaling in RAW 264.7 macrophages	[153]
**118**	from various species of Ochrophyta	ABTS^+^ scavenging: 72.06 ± 0.70% inhibition at 2 mM β-carotene bleaching: 95% inhibition at 150 μg/mL DPPH scavenging: IC_50_ = 19.6, 206.4 μM Fe^2+^ chelation: IC_50_ = 1.52 mM FRAP: 15.2 μg TE; 24.62 mg ascorbic acid eqs/g at 1.5 mM OH scavenging: IC_50_ = 51.6 μM O_2_^−^ scavenging ROO scavenging (ORAC/ESR) caspase-3/7 activation high glucose-induced oxidative stress in HUVEC and zebrafish model H_2_O_2_-induced intracellular ROS and cytotoxicity in fibroblast cells H_2_O_2_-induced neuronal apoptosis in SH-SY5Y cells intracellular ROS generation in SH-SY5Y cells (DCFH-DA) LPS- and IFN-γ-induced NO generation and Nrf2/ARE signaling in RAW 264.7 macrophages oxidative DNA damage PI3-K/Akt cascade/ERK signaling square wave voltammetry TPA-induced O_2_^−^ generation in differentiated HL-60 cells	[142,153,154,155,156,157,158,159,160,161,162]
**119**	*Laminaria japonica* (Ochrophyta, Phaeophyceae, Laminariales)	ABTS^+^ scavenging DPPH scavenging OH scavenging O_2_^−^ scavenging	[162]
**120**	*L. japonica* (Ochrophyta, Phaeophyceae, Laminariales)	ABTS^+^ scavenging DPPH scavenging OH scavenging O_2_^−^ scavenging	[162]
**121**	*L. japonica* (Ochrophyta, Phaeophyceae, Laminariales)	ABTS^+^ scavenging DPPH scavenging OH scavenging O_2_^−^ scavenging	[162]
**122**	from plants and microalgae, but also from macroalgae	ABTS^+^ scavenging: IC_50_ = 25.4 μM DPPH scavenging: IC_50_ = 68.9 μM	[163]
**123**	*Undariopsis peterseniana* (Ochrophyta, Phaeophyceae, Laminariales)	oxidative stress-mediated apoptosis	[164]
**124**	*Sargassum horneri* (Ochrophyta, Phaeophyceae, Fucales)	alkyl scavenging (ESR): IC_50_: 0.22 ± 0.02 mM AAPH-induced intracellular ROS in Vero cells AAPH-induced lipid peroxidation in zebrafish models in vivo NF-κB, MAPK and oxidative stress regulation in RAW 264.7 macrophages Nrf2/HO-1 pathways regulation	[165,166]

AAPH: 2,2′-azobis(2-amidinopropane) dihydrochloride; ABTS^+^: 2,2’-azino-bis (3-ethyl benzothiazoline-6-sulfonic acid) diammonium salt; Akt: protein kinase B; ARE: antioxidant response element; CAT: catalase; DCFH-DA: cell-based 2′,7′-dichlorodihydrofluorescein diacetate antioxidant assay; DPPH: 1,1-diphenyl-2-picrylhydrazyl free radical; ESR: electron spin resonance; FRAP: ferric reducing antioxidant power; GSH: glutathione; GPx: glutathione peroxidase; HO-1: heme oxygenase-1; H_2_O_2_: hydrogen peroxide; HUVEC: human umbilical vein endothelial cells; OH: hydroxyl; IC_50_: half maximal inhibitory concentration; IFN-γ: interferon γ; LPS: lipopolysaccharide; MAPK: mitogen-activated protein kinase; NADPH: nicotinamide adenine dinucleotide phosphate; NF-κB: nuclear factor kappa-light-chain-enhancer of activated B cells, NO.: nitric oxide; Nox: NADPH oxidase; Nrf2: nuclear factor erythroid 2-related factor 2; O_2_^−^: superoxide anion; ORAC: oxygen radical absorbance capacity; PI3-K: phosphatidylinositol 3-kinase; ROS: reactive oxygen species; SH-SY5Y: human dopaminergic neuronal cell line; SOD: superoxide dismutase; TBARS: thiobarbituric acid reactive substances; t-BHP: *tert*-butyl hydroperoxide; TE: trolox equivalents; TPA: 12-*O*-tetradecanoylphorbol 13-acetate; XO: xanthine oxidase.

**Table 4:** Meroterpenoids from macroalgae with antioxidant activity.

Compound	Isolation Source	Assay/Activity	Reference
**125**	*Cymopolia barbata* (Chlorophyta, Ulvophyceae, Dasycladales)	DPPH scavenging: strong exogenous ROS scavenging in TPA-treated HL-60 cells (DCFH-DA): IC_50_ = 4.0 μM	[91]
**126**	*C. barbata* (Chlorophyta, Ulvophyceae, Dasycladales)	DPPH scavenging: strong exogenous ROS scavenging in TPA-treated HL-60 cells (DCFH-DA): IC_50_ >14.6 μM	[91]
**127**	*Cystoseira crinita* (Ochrophyta, Phaeophyceae, Fucales)	ABTS^+^ scavenging DPPH scavenging: 94.1% at 230 μM O_2_^−^ generation (PCL assay) TBARS assay: 66.8% inhibition at 164 μM	[177]
**128**	*C. crinita* (Ochrophyta, Phaeophyceae, Fucales)	ABTS^+^ scavenging DPPH scavenging activity: 92.5% at 230 μM O_2_^−^ generation (PCL assay) TBARS assay: 66.5% inhibition at 164 μM	[177]
**129**	*Cystoseira usneoides* (Ochrophyta, Phaeophyceae, Fucales)	ABTS^+^ scavenging: IC_50_ = 33.3 ± 2.3 μM; 0.78 TE	[178]
**130**	*C. usneoides* (Ochrophyta, Phaeophyceae, Fucales)	ABTS^+^ scavenging: IC_50_ = 51.6 ± 4.8 μM; 0.50 TE	[178]
**131**	*C. usneoides* (Ochrophyta, Phaeophyceae, Fucales)	ABTS^+^ scavenging: IC_50_ = 44.7 ± 1.1 μM; 0.58 TE	[178]
**132**	*C. usneoides* (Ochrophyta, Phaeophyceae, Fucales)	ABTS^+^ scavenging: IC_50_ = 55.9 ± 9.9 μM; 0.46 TE	[178]
**133**	*Dictyopteris undulata* (Ochrophyta, Phaeophyceae, Dictyotales)	DPPH scavenging: IC_50_ = 71 μM	[179]
**134**	*D. undulata* (Ochrophyta, Phaeophyceae, Dictyotales)	expression of phase-2 enzymes (i.e., NQO1, GSH S-transferase, HO-1 and PRDX4) Nrf2/ARE signaling pathway oxidative stress in HT22 hippocampal neuronal cells	[180]
**135**	*D. undulata* (Ochrophyta, Phaeophyceae, Dictyotales)	DPPH scavenging: IC_50_ = 121 μM	[179]
**136**	*G. salicornia* (Rhodophyta, Florideophyceae, Gracilariales)	ABTS^+^ scavenging: IC_50_ = 1.88 ± 0.02 mM DPPH scavenging: IC_50_ = 1.51 ± 0.01 mM	[181]
**137**	*G. salicornia* (Rhodophyta, Florideophyceae, Gracilariales)	ABTS^+^ scavenging: IC_50_ = 1.96 ± 0.01 mM DPPH scavenging: IC_50_ = 1.85 ± 0.02 mM	[181]
**138**	*G. salicornia* (Rhodophyta, Florideophyceae, Gracilariales)	ABTS^+^ scavenging: IC_50_ = 1.57 ± 0.02 mM DPPH scavenging: IC_50_ = 1.33 ± 0.01 mM	[181]
**139**	*D. undulata* (Ochrophyta, Phaeophyceae, Dictyotales)	DPPH scavenging: IC_50_ = 145 μM	[179]
**140**	*G. salicornia* (Rhodophyta, Florideophyceae, Gracilariales)	ABTS^+^ scavenging: IC_50_ = 1.50 mM DPPH scavenging: IC_50_ = 1.40 mM	[182]
**141**	*G. salicornia* (Rhodophyta, Florideophyceae, Gracilariales)	ABTS^+^ scavenging: IC_50_ = 1.33 mM DPPH scavenging: IC_50_ = 1.17 mM	[182]
**142**	*S. micracanthum* (Ochrophyta, Phaeophyceae, Fucales)	DPPH scavenging: IC_50_ = 25.5 μM lipid peroxidation in rat liver: IC_50_ = 0.26 μM	[183]
**143**	*S. micracanthum* (Ochrophyta, Phaeophyceae, Fucales)	DPPH scavenging: 3.0% at 0.23 mM lipid peroxidation in rat liver: IC_50_ = 2.22 μM	[184]
**144**	*Cystoseira abies-marina* (Ochrophyta, Phaeophyceae, Fucales)	DPPH scavenging: 29% at 1.06 mM	[185]
**145**	*C. abies-marina* (Ochrophyta, Phaeophyceae, Fucales)	DPPH scavenging: 30% at 1.02 mM	[185]
**146**	*C. crinita* (Ochrophyta, Phaeophyceae, Fucales)	ABTS^+^ scavenging DPPH scavenging: 94.4% at 230 μM O_2_^−^ radical generation (PCL assay) TBARS: 70.8% inhibition at 164 μM	[177]
**147**	*C. crinita* (Ochrophyta, Phaeophyceae, Fucales)	ABTS^+^ scavenging: TEAC = 0.14 mM DPPH scavenging: 95.4% at 230 μM O_2_^−^ radical generation (PCL assay): 1.35 TBARS: 71.8% inhibition at 164 μM	[177]
**148**	*C. crinita* (Ochrophyta, Phaeophyceae, Fucales)	ABTS^+^ scavenging DPPH scavenging: 96.1% at 230 μM O_2_^−^ radical generation (PCL assay) TBARS: 68.9% inhibition at 164 μM	[177]
**149**	*C. crinita* (Ochrophyta, Phaeophyceae, Fucales)	ABTS^+^ scavenging DPPH scavenging: 95.5% at 230 μM O_2_^−^ radical generation (PCL assay) TBARS: 70.3% inhibition at 164 μM	[177]
**150**	*C. crinita* (Ochrophyta, Phaeophyceae, Fucales)	ABTS^+^ scavenging: TEAC = 0.37 mM DPPH scavenging: 95.5% at 230 μM O_2_^−^ radical generation (PCL assay): 1.39 TBARS: 72.2% inhibition at 164 μM	[177]
**151**	*C. crinita* (Ochrophyta, Phaeophyceae, Fucales)	ABTS^+^ scavenging: TEAC = 0.09 mM DPPH scavenging: 95.7% at 230 μM O_2_^−^ radical generation (PCL assay): 0.72 TBARS: 71.1% inhibition at 164 μM	[177]
**152**	*C. crinita* (Ochrophyta, Phaeophyceae, Fucales)	ABTS^+^ scavenging: TEAC = 0.09 mM DPPH scavenging: 96.4% at 230 μM O_2_^−^ radical generation (PCL assay): 0.59 TBARS: 73.7% inhibition at 164 μM	[177]
**153**	*C. crinita* (Ochrophyta, Phaeophyceae, Fucales)	ABTS^+^ scavenging: TEAC = 0.09 mM DPPH scavenging: 96.7% at 230 μM O_2_^−^ radical generation (PCL assay): 0.51 TBARS: 73.4% inhibition at 164 μM	[177]
**154**	*C. crinita* (Ochrophyta, Phaeophyceae, Fucales)	ABTS^+^ scavenging: TEAC = 0.08 mM DPPH scavenging: 65.4% at 230 μM O_2_^−^ radical generation (PCL assay): 1.06 TBARS: 74.9% inhibition at 164 μM	[177]
**155**	*C. crinita* (Ochrophyta, Phaeophyceae, Fucales)	ABTS^+^ scavenging: TEAC = 0.28 mM DPPH scavenging: 95.8% at 230 μM O_2_^−^ radical generation (PCL assay): 0.79 TBARS: 74.6% inhibition at 164 μM	[177]
**156**	*C. usneoides* (Ochrophyta, Phaeophyceae, Fucales)	ABTS^+^ scavenging: 0.77 TE	[186]
**157**	*C. usneoides* (Ochrophyta, Phaeophyceae, Fucales)	ABTS^+^ scavenging: IC_50_ = 24.5 ± 1.6 μM; 1.06 TE	[178]
**158**	*C. usneoides* (Ochrophyta, Phaeophyceae, Fucales)	ABTS^+^ scavenging: 0.77 TE	[186]
**159**	*C. usneoides* (Ochrophyta, Phaeophyceae, Fucales)	ABTS^+^ scavenging: IC_50_ = 26.3 ± 2.3 μM; 0.98 TE	[178]
**160**	*C. usneoides* (Ochrophyta, Phaeophyceae, Fucales)	ABTS^+^ scavenging: 0.87 TE	[186]
**161**	*C. usneoides* (Ochrophyta, Phaeophyceae, Fucales)	ABTS^+^ scavenging: IC_50_ = 33.1 ± 5.1 μM; 0.78 TE	[178]
**162**	*C. usneoides* (Ochrophyta, Phaeophyceae, Fucales)	ABTS^+^ scavenging: 0.67 TE	[186]
**163**	*C. usneoides* (Ochrophyta, Phaeophyceae, Fucales)	ABTS^+^ scavenging: 0.81 TE	[186]
**164**	*C. usneoides* (Ochrophyta, Phaeophyceae, Fucales)	ABTS^+^ scavenging: IC_50_ = 43.1 ± 3.1 μM; 0.60 TE	[178]
**165**	*C. usneoides* (Ochrophyta, Phaeophyceae, Fucales)	ABTS^+^ scavenging: 0.53 TE	[186]
**166**	*C. usneoides* (Ochrophyta, Phaeophyceae, Fucales)	ABTS^+^ scavenging: 0.37 TE	[186]
**167**	*C. usneoides* (Ochrophyta, Phaeophyceae, Fucales)	ABTS^+^ scavenging: 0.66 TE	[186]
**168**	*C. usneoides* (Ochrophyta, Phaeophyceae, Fucales)	ABTS^+^ scavenging: 0.45 TE	[186]
**169**	*C. usneoides* (Ochrophyta, Phaeophyceae, Fucales)	ABTS^+^ scavenging: 0.65 TE	[186]
**170**	*C. usneoides* (Ochrophyta, Phaeophyceae, Fucales)	ABTS^+^ scavenging: 0.50 TE	[186]
**171**	*C. usneoides* (Ochrophyta, Phaeophyceae, Fucales)	ABTS^+^ scavenging: 0.62 TE	[186]
**172**	*C. usneoides* (Ochrophyta, Phaeophyceae, Fucales)	ABTS^+^ scavenging: IC_50_ = 24.4 ± 0.9 μM; 1.06 TE	[178]
**173**	*C. usneoides* (Ochrophyta, Phaeophyceae, Fucales)	ABTS^+^ scavenging: IC_50_ = 22.5 ± 2.1 μM; 1.15 TE	[178]
**174**	*Sargassum siliquastrum* (Ochrophyta, Phaeophyceae, Fucales)	DPPH scavenging: IC_50_ = 0.54 μM	[187]
**175**	*Sargassum elegans*, *S. siliquastrum*, *Sargassum thunbergii* (Ochrophyta, Phaeophyceae, Fucales)	DPPH scavenging: IC_50_ = 0.40; 46.9 μM ONOO scavenging: 78.03% at 23.4 μM ONOO^−^ derived from SIN-1 scavenging: 100% at 23.4 μM electrochemistry-guided isolation of antioxidant metabolites (using square wave and cyclic voltammetry methods)	[157,187,188,189]
**176**	*S. micracanthum* (Ochrophyta, Phaeophyceae, Fucales)	DPPH scavenging: 52.6% inhibition at 143.6 μM lipid peroxidation in rat liver: IC_50_ = 63.6 μM	[184]
**177**	*S. micracanthum* (Ochrophyta, Phaeophyceae, Fucales)	DPPH scavenging: 32.3% inhibition at 144.0 μM lipid peroxidation in rat liver: IC_50_ = 1.66 μM	[184]
**178**	*S. siliquastrum* (Ochrophyta, Phaeophyceae, Fucales)	DPPH scavenging: IC_50_ = 0.27 μM	[187]
**179**	*S. siliquastrum* (Ochrophyta, Phaeophyceae, Fucales)	DPPH scavenging: IC_50_ = 0.25 μM	[187]
**180**	*S. siliquastrum* (Ochrophyta, Phaeophyceae, Fucales)	DPPH scavenging: IC_50_ = 0.68 μM	[187]
**181**	*S. siliquastrum* (Ochrophyta, Phaeophyceae, Fucales)	DPPH scavenging: IC_50_ = 0.64 μM	[187]
**182**	*S. siliquastrum* (Ochrophyta, Phaeophyceae, Fucales)	DPPH scavenging: IC_50_ = 0.62 μM	[187]
**183**	*S. siliquastrum* (Ochrophyta, Phaeophyceae, Fucales)	DPPH scavenging: IC_50_ = 0.21 μM	[187]
**184**	*S. siliquastrum* (Ochrophyta, Phaeophyceae, Fucales)	DPPH scavenging: IC_50_ = 23.3 μM	[187]
**185**	*S. siliquastrum* (Ochrophyta, Phaeophyceae, Fucales)	DPPH scavenging: IC_50_ = 26.1 μM	[187]
**186**	*S. siliquastrum* (Ochrophyta, Phaeophyceae, Fucales)	DPPH scavenging: IC_50_ = 25.4 μM	[187]
**187**	*S. siliquastrum* (Ochrophyta, Phaeophyceae, Fucales)	DPPH scavenging: IC_50_ = 37.9 μM	[187]
**188**	*S. siliquastrum* (Ochrophyta, Phaeophyceae, Fucales)	DPPH scavenging: IC_50_ = 35.4 μM	[187]
**189**	*S. siliquastrum* (Ochrophyta, Phaeophyceae, Fucales)	DPPH scavenging: IC_50_ = 18.7 μM	[187]
**190**	*S. siliquastrum* (Ochrophyta, Phaeophyceae, Fucales)	DPPH scavenging: IC_50_ = 25.9 μM	[187]
**191**	*S. siliquastrum* (Ochrophyta, Phaeophyceae, Fucales)	DPPH scavenging: IC_50_ = 30.4 μM	[187]
**192**	*S. siliquastrum* (Ochrophyta, Phaeophyceae, Fucales)	DPPH scavenging: IC_50_ = 47.9 μM	[187]
**193**	*S. siliquastrum* (Ochrophyta, Phaeophyceae, Fucales)	DPPH scavenging: IC_50_ = 26.3 μM	[187]
**194**	*S. siliquastrum* (Ochrophyta, Phaeophyceae, Fucales)	DPPH scavenging: IC_50_ = 25.1 μM	[187]
**195**	*S. micracanthum* (Ochrophyta, Phaeophyceae, Fucales)	DPPH scavenging: IC_50_ = 933.3 μM lipid peroxidation in rat liver: IC_50_ = 2.33 μM	[183]
**196**	*S. elegans* (Ochrophyta, Phaeophyceae, Fucales)	electrochemistry-guided isolation of antioxidant metabolites (using square wave and cyclic voltammetry methods)	[157]
**197**	*S. elegans*, *S. micracanthum*, *S. thunbergii* (Ochrophyta, Phaeophyceae, Fucales)	DPPH scavenging: IC_50_ = 63.6; 100.2 μM; 69.82% at 250 μM ONOO scavenging: 64.18% at 23.6 μM ONOO^−^ derived from SIN-1 scavenging activity: 75.39% at 23.6 μM electrochemistry-guided isolation of antioxidant metabolites (using square wave and cyclic voltammetry methods)	[157,188,189,190]
**198**	*C. crinita* (Ochrophyta, Phaeophyceae, Fucales)	ABTS^+^ scavenging DPPH scavenging: 29.0% at 230 μM O_2_^−^ generation (PCL assay) TBARS: 43.3% inhibition at 164 μM	[177]
**199**	*C. crinita* (Ochrophyta, Phaeophyceae, Fucales)	ABTS^+^ scavenging: TEAC = 0.30 mM DPPH scavenging: 38.6% at 230 μM O_2_^−^ generation (PCL assay): 1.41 TBARS: 54.4% inhibition at 164 μM	[177]
**200**	*C. barbata* (Ochrophyta, Phaeophyceae, Fucales)	antioxidant activity against ROS and reactive nitrogen species	[141,183,184,189]
**201**	*S. siliquastrum* (Ochrophyta, Phaeophyceae, Fucales)	DPPH scavenging: 90.0% at 0.29 mM	[191]
**202**	*S. siliquastrum* (Ochrophyta, Phaeophyceae, Fucales)	DPPH scavenging: 87.4% at 0.29 mM	[191]
**203**	*S. siliquastrum* (Ochrophyta, Phaeophyceae, Fucales)	H_2_O_2_-induced lipid peroxidation in HT 1080 cells intracellular GSH level in HT 1080 cells intracellular ROS generation (DCFH-DA) in HT 1080 cells	[192]
**204**	*S. siliquastrum* (Ochrophyta, Phaeophyceae, Fucales)	DPPH scavenging: 90.5% at 0.24 mM	[191]
**205**	*S. siliquastrum* (Ochrophyta, Phaeophyceae, Fucales)	DPPH scavenging: 89.6% at 0.23 mM H_2_O_2_-induced lipid peroxidation in HT 1080 cells intracellular GSH level in HT 1080 cells intracellular ROS generation (DCFH-DA) in HT 1080 cells: 67.2% decrease at 11.7 μM	[191,192]
**206**	*S. siliquastrum* (Ochrophyta, Phaeophyceae, Fucales)	DPPH scavenging: 87.3% at 0.23 mM H_2_O_2_-induced lipid peroxidation in HT 1080 cells intracellular GSH level in HT 1080 cells intracellular ROS generation (DCFH-DA) in HT 1080 cells: 87.2% decrease at 11.7 μM	[191,192]
**207**	*S. siliquastrum* (Ochrophyta, Phaeophyceae, Fucales)	DPPH scavenging: 88.2% at 0.23 mM	[191]
**208**	*S. siliquastrum* (Ochrophyta, Phaeophyceae, Fucales)	DPPH scavenging: 90.4% at 0.23 mM expression of osteoclastic marker gene in RANKL-stimulated RAW264.7 cells (TRAP, CTSK, MMP9 and CTR) NF-κB activation in RANKL-stimulated RAW264.7 cells osteoclast differentiation in RANKL-stimulated RAW264.7 cells phosphorylation of MAPKs in RANKL-stimulated RAW264.7 cells	[191,193]
**209**	*S. siliquastrum* (Ochrophyta, Phaeophyceae, Fucales)	DPPH scavenging: 89.2% at 0.23 mM H_2_O_2_-induced lipid peroxidation in HT 1080 cells intracellular GSH level in HT 1080 cells intracellular ROS generation (DCFH-DA assay) in HT 1080 cells	[191,192]
**210**	*S. siliquastrum* (Ochrophyta, Phaeophyceae, Fucales)	DPPH scavenging: 87.8% at 0.23 mM	[191]
**211**	*S. siliquastrum* (Ochrophyta, Phaeophyceae, Fucales)	DPPH scavenging: 90.4% at 0.23 mM	[191]
**212**	*S. siliquastrum* (Ochrophyta, Phaeophyceae, Fucales)	DPPH scavenging: 89.1% at 0.23 mM	[191]
**213**	*S. micracanthum* (Ochrophyta, Phaeophyceae, Fucales)	NADPH-dependent lipid peroxidation in rat microsomes: IC_50_ = 0.65 μM	[194]
**214**	*S. micracanthum*, *S. thunbergii* (Ochrophyta, Phaeophyceae, Fucales)	DPPH scavenging: IC_50_ = 75.4 μM; 78.85% at 250 μM ONOO scavenging: 92.69% at 23.6 μM ONOO^−^ derived from SIN-1 scavenging: 99.51% at 23.6 μM	[188,189,190]
**215**	*S. thunbergii* (Ochrophyta, Phaeophyceae, Fucales)	DPPH scavenging: IC_50_ = 82.9 μM	[189]
**216**	*S. siliquastrum* (Ochrophyta, Phaeophyceae, Fucales)	H_2_O_2_-induced lipid peroxidation in HT 1080 cells: 43.2% at 112.0 μM intracellular GSH level in HT 1080 cells intracellular ROS generation (DCFH-DA) in HT 1080 cells	[192]
**217**	*S. siliquastrum* (Ochrophyta, Phaeophyceae, Fucales)	H_2_O_2_-induced lipid peroxidation in HT 1080 cells: 38.9% at 112.0 μM intracellular GSH level in HT 1080 cells intracellular ROS generation (DCFH-DA) in HT 1080 cells	[192]
**218**	*S. siliquastrum* (Ochrophyta, Phaeophyceae, Fucales)	DPPH scavenging: 88.8% at 0.24 mM	[191]
**219**	*S. thunbergii* (Ochrophyta, Phaeophyceae, Fucales)	DPPH scavenging: IC_50_ = 67.8 μM ONOO scavenging: 60.0% at 11.3 μM ONOO^−^ derived from SIN-1 scavenging: 98.6% at 11.3 μM	[195]
**220**	*S. thunbergii* (Ochrophyta, Phaeophyceae, Fucales)	DPPH scavenging: IC_50_ = 70.0 μM ONOO scavenging: 57.1% at 11.3 μM ONOO^−^ derived from SIN-1 scavenging: 90.6% at 11.3 μM	[195]
**221**	*S. siliquastrum* (Ochrophyta, Phaeophyceae, Fucales)	DPPH scavenging: 90.1% at 0.24 mM	[191]
**222**	*S. siliquastrum* (Ochrophyta, Phaeophyceae, Fucales)	DPPH scavenging: 88.7% at 0.23 mM	[191]
**223**	*S. siliquastrum* (Ochrophyta, Phaeophyceae, Fucales)	DPPH scavenging: 89.2% at 0.24 mM	[191]
**224**	*S. siliquastrum* (Ochrophyta, Phaeophyceae, Fucales)	DPPH scavenging: 88.7% at 0.24 mM	[191]

ABTS^+^: 2,2’-azino-bis (3-ethyl benzothiazoline-6-sulfonic acid) diammonium salt; ARE: antioxidant response element; CTR: calcitonin receptor; CTSK: cathepsin K; DCFH-DA: cell-based 2′,7′-dichlorodihydrofluorescein diacetate antioxidant assay; DPPH: 1,1-diphenyl-2-picrylhydrazyl free radical; GSH: glutathione; HO-1: heme oxygenase-1; HT 1080: human fibrosarcoma cell line; IC_50_: half maximal inhibitory concentration; MMP9: matrix metalloproteinase 9; NADPH: nicotinamide adenine dinucleotide phosphate; NQO1: NADPH quinone oxidoreductase 1; Nrf2: nuclear factor erythroid 2-related factor 2; ONOO^−^: peroxynitrite; O_2_^−^: superoxide anion; PCL: photochemiluminescence; PRDX4: peroxyredoxin 4; RANKL: receptor activator of NF-κB ligand; ROS: reactive oxygen species; SIN-1: 3-morpholinosydnonimine; SOD: superoxide dismutase; TBARS: thiobarbituric acid reactive substances; TE: trolox equivalents; TEAC: trolox equivalence antioxidant capacity; TPA: 12-*O*-tetradecanoylphorbol 13-acetate; TRAP: tartrate-resistant acid phosphatase.

**Table 5:** Nitrogenous compounds from macroalgae with antioxidant activity.

Compound	Isolation Source	Assay/Activity	Reference
**225**	*Porphyra yezoensis* (Rhodophyta, Bangiophyceae, Bangiales)	DPPH scavenging: IC_50_ = 185.2 ± 3.2 μM ORAC: 51 ± 7% TE Nrf2-regulated antioxidant response in UVA-treated fibroblasts (1BR)	[196,197]
**226**	*G. furcata* (Rhodophyta, Florideophyceae, Gigartinales)	DPPH scavenging: IC_50_ = 399.0 ± 1.1 μM ORAC: 17 ± 7% TE Nrf2-regulated antioxidant response in UVA-treated fibroblasts (1BR)	[196]
**227**	*P. yezoensis* (Rhodophyta, Bangiophyceae, Bangiales)	DPPH scavenging: IC_50_ = 30.8 μM	[197]
**228**	*P. yezoensis* (Rhodophyta, Bangiophyceae, Bangiales)	TBARS: 85.2% inhibition FTC: 84.1% inhibition	[198]
**229**	*P. yezoensis* (Rhodophyta, Bangiophyceae, Bangiales)	TBARS: 94.4% inhibition FTC: 89.1% inhibition	[198]
**230**	*Martensia fragilis* (Rhodophyta, Florideophyceae, Ceramiales)	DPPH scavenging: moderate exogenous ROS scavenging in TPA-treated HL-60 cells (DCFH-DA): IC_50_ = 11 μM	[91]
**231**	*Dictyota coriacea* (Ochrophyta, Phaeophyceae, Dictyotales)	H_2_O_2_-induced oxidative damage and toxicity in neuron-like PC12 cell Nrf2/ARE signaling pathway	[199]
**232**	*Porphyra dioica* (Rhodophyta, Bangiophyceae, Bangiales)	ORAC: 3.79 ± 0.11 μmol TE/μM	[200]
**233**	*P. dioica* (Rhodophyta, Bangiophyceae, Bangiales)	ORAC: 3.14 ± 0.32 μmol TE/μM	[200]
**234**	*P. dioica* (Rhodophyta, Bangiophyceae, Bangiales)	ORAC: 0.09 ± 0.00 μmol TE/μM	[200]
**235**	*P. dioica* (Rhodophyta, Bangiophyceae, Bangiales)	ORAC: 2.85 ± 0.42 μmol TE/μM	[200]
**236**	*P. dioica* (Rhodophyta, Bangiophyceae, Bangiales)	ORAC: 2.50 ± 0.16 μmol TE/μM	[200]
**237**	*P. dioica* (Rhodophyta, Bangiophyceae, Bangiales)	ORAC: 4.27 ± 0.15 μmol TE/μM	[200]
**238**	*P. dioica* (Rhodophyta, Bangiophyceae, Bangiales)	ORAC: 0.92 ± 0.10 μmol TE/μM	[200]
**239**	*Porphyra* sp. (Rhodophyta, Bangiophyceae, Bangiales)	ROO scavenging (CBA): 0.048 ± 0.003 mmol TE/g	[201]
**240**	*Enteromorpha prolifera* (Chlorophyta, Ulvophyceae, Ulvales)	DPPH scavenging: 88.6 ± 1.3% at 168.7 μM reducing power: 60% at 843.6 μM ROO scavenging: 50% at 843.6 μM TPC: 21.4 ± 0.1 mg GAE/g	[202]
**241**	from plants and microalgae, but also from macroalgae	β-carotene bleaching: 49.63% at 56.0 μM DPPH scavenging: 13.89% at 56.0 μM Fe^2+^ chelation: 55% at 200 μM lipid peroxidation: 95% at 100 μM ROO scavenging capacity: 308	[141,203,204,205]
**242**	*E. bicyclis* (Ochrophyta, Phaeophyceae, Laminariales)	FTC TBARS	[206]

CBA: crocin bleaching activity; DPPH: 1,1-diphenyl-2-picrylhydrazyl free radical; FTC: ferric thiocyanate; GAE: gallic acid equivalents; Nrf2: nuclear factor erythroid 2-related factor 2; ORAC: oxygen radical absorbance capacity; ROO: peroxyl; TBARS: thiobarbituric acid reactive substances; TE: trolox equivalents; TPC: total phenolics content.

**Table 6:** Carbohydrates and polysaccharides from macroalgae with antioxidant activity.

Compound	Isolation Source	MW/Sulfate Content	Assay/Activity	Reference
**243**	from a plethora of macroalgae	-	free radicals (DPPH, OH, NO, O_2,_) scavenging enzyme activity (a-glucosidase, AChE, BChE)	[210]
**244**	*Laurencia undulata* (Rhodophyta, Florideophyceae, Ceramiales)	-	alkyl scavenging: IC_50_ = 43.7 μM DPPH scavenging: IC_50_ = 39.3 μM OH scavenging: IC_50_ = 27.4 μM O_2_^−^ scavenging: IC_50_ = 39.4 μM gene expression levels of GSH and SOD intracellular ROS levels (DCFH-DA) in RAW264.7 cells membrane protein oxidation MPO activity protein expression of MMP2 and MMP9	[211]
**245**	*L. undulata* (Rhodophyta, Florideophyceae, Ceramiales)	-	alkyl scavenging: IC_50_ = 32.3 μM DPPH scavenging: IC_50_ = 41.8μM OH scavenging: IC_50_ = 22.7 μM O_2_^−^ scavenging: IC_50_ = 33.6 μM gene expression levels of GSH and SOD intracellular ROS levels (DCFH-DA) in RAW264.7 cells membrane protein oxidation MPO activity protein expression of MMP2 and MMP9	[211]
**246**	enzymatically produced from commercially available polysaccharides	n.d.	OH scavenging O_2_^−^ scavenging erythrocyte hemolysis inhibiting lipid peroxidation metal chelating activity	[212]
**247**	enzymatically produced from commercially available polysaccharides	n.d.	OH scavenging O_2_^−^ scavenging erythrocyte hemolysis inhibiting lipid peroxidation metal chelating activity	[212]
**248**	*F. vesiculosus* (Ochrophyta, Phaeophyceae, Fucales)	170 kDa/44.10 ± 0.16%	OH scavenging: IC_50_ = 0.157 ± 0.005 mg/mL O_2_^−^ scavenging: IC_50_ = 0.058 ± 0.011 mg/mL liver microsomal lipid peroxidation: IC_50_ = 1.250 ± 0.174 mg/mL	[213]
**249**	*Cystoseira sedoides* (Ochrophyta, Phaeophyceae, Fucales)	642 kDa/16.3%	DPPH scavenging: IC_50_ = 0.96 ± 0.01 mg/mL	[214]
**250**	*Cystoseira compressa* (Ochrophyta, Phaeophyceae, Fucales)	545 kDa/16.6%	DPPH scavenging: IC_50_ = 0.84 ± 0.06 mg/mL	[214]
**251**	*C. crinita* (Ochrophyta, Phaeophyceae, Fucales)	339 kDa/15.7%	DPPH scavenging: IC_50_ = 0.76 ± 0.04 mg/mL	[214]
**252**	*Padina gymnospora* (Ochrophyta, Phaeophyceae, Dictyotales)	200 kDa/18.40 ± 0.28%	OH scavenging O_2_^−^ scavenging: IC_50_ = 0.243 ± 0.014 mg/mL liver microsomal lipid peroxidation: IC_50_ = 2.753 ± 0.051 mg/mL	[213]
**253**	*P. gymnospora* (Ochrophyta, Phaeophyceae, Dictyotales)	18 kDa/27.57 ± 0.17%	OH scavenging: IC_50_ = 0.353 ± 0.036 mg/mL O_2_^−^ scavenging: IC_50_ = 0.243 ± 0.013 mg/mL liver microsomal lipid peroxidation: IC_50_ = 23.887 ± 5.975 mg/mL	[213]
**254**	*L. japonica* (Ochrophyta, Phaeophyceae, Laminariales)	742 kDa/16.5%	OH scavenging: IC_50_ = 0.60 mg/mL O_2_^−^ scavenging: IC_50_ = 0.43 mg/mL	[215]
**255**	*L. japonica* (Ochrophyta, Phaeophyceae, Laminariales)	175.9 kDa/33.5%	OH scavenging: IC_50_ = 0.85 mg/mL O_2_^−^ scavenging: IC_50_ = 0.53 mg/mL	[215]
**256**	*Undaria pinnatifida* (Ochrophyta, Phaeophyceae, Laminariales)	10 kDa/n.d.	DPPH scavenging: 8.77 ± 1.24 TE (μg/mL) OH scavenging: 86.98 ± 1.16%	[216]
**257**	*U. pinnatifida* (Ochrophyta, Phaeophyceae, Laminariales)	300 kDa/20.01 ± 0.82%	DPPH scavenging: 9.01 ± 1.93 TE (μg/mL) OH scavenging: 74.32 ± 1.41%	[216]
**258**	*F. vesiculosus* (Ochrophyta, Phaeophyceae, Fucales)	n.d./21.1 ± 1.7%	ABTS^+^ scavenging DPPH scavenging lipid oxidation differential pulse voltammetry	[217]
**259**	*F. vesiculosus* (Ochrophyta, Phaeophyceae, Fucales)	n.d./21.2 ± 0.8%	ABTS^+^ scavenging DPPH scavenging lipid oxidation differential pulse voltammetry	[217]
**260**	*F. vesiculosus* (Ochrophyta, Phaeophyceae, Fucales)	n.d./27.0%	DPPH scavenging: IC_50_ = 0.035 ± 0.002 mg/mL reducing power: RC_0_._5AU_ = 1.48 mg/mL	[218]
**261**	*Sargassum binderi* (Ochrophyta, Phaeophyceae, Fucales)	n.d./n.d.	DPPH scavenging: IC_50_ = 2.01 ± 0.29 mg/mL OH scavenging: 60.95 ± 0.69% O_2_^−^ scavenging: 26.78 ± 1.90% reducing power: 0.60 ± 0.08 mg GAE/100 g	[219]
**262**	hydrolyzed from commercially available polysaccharides	5–30 kDa/n.d.	LPS-induced ROS generation in RAW 264.7 macrophages	[220]
**263**	not specified	n.d./n.d.	HO-1, SOD1, Nrf2 and Keap1 expression in human keratinocytes	[221]
**264**	*U. pinnatifida* (Ochrophyta, Phaeophyceae, Laminariales)	n.d./n.d.	DPPH scavenging metal chelating activity NO scavenging OH scavenging reducing power arthritis-induced physical changes in rats	[222]
**265**	*Eucheuma spinosa* (Rhodophyta, Florideophyceae, Gigartinales)	n.d./27.60 ± 0.12%	OH scavenging: IC_50_ = 0.281 ± 0.072 mg/mL O_2_^−^ scavenging: IC_50_ = 0.332 ± 0.080 mg/mL liver microsomal lipid peroxidation: IC_50_ = 0.830 ± 0.063 mg/mL	[213]
**266**	*Eucheuma cottonii* (Rhodophyta, Florideophyceae, Gigartinales)	n.d./17.90 ± 0.05%	OH scavenging: IC_50_ = 0.335 ± 0.016 mg/mL O_2_^−^ scavenging: IC_50_ = 0.112 ± 0.003 mg/mL liver microsomal lipid peroxidation: IC_50_ = 0.323 ± 0.011 mg/mL	[213]
**267**	*Gigartina acicularis, Gigartina pisillata* (Rhodophyta, Florideophyceae, Gigartinales)	n.d./33.38 ± 0.06%	OH scavenging: IC_50_ = 0.357 ± 0.120 mg/mL O_2_^−^ scavenging: IC_50_ = 0.046 ± 0.001 mg/mL liver microsomal lipid peroxidation: IC_50_ = 2.697 ± 0.267 mg/mL	[213]
**268**	*Porphyra haitanensis* (Rhodophyta, Bangiophyceae, Bangiales)	n.d./17.7%	OH scavenging: IC_50_ = 6.55 mg/mL O_2_^−^ scavenging: ~60% at 2.5 μg/mL reducing power: 0.42 at 6.17 mg/mL	[223]
**269**	*Ulva pertusa* (Chlorophyta, Ulvophyceae, Ulvales)	n.d./19.5%	OH scavenging O_2_^−^ scavenging: IC_50_ = 20.0 μg/mL metal chelating assay reducing power	[224]
**270**	*U. pertusa* (Chlorophyta, Ulvophyceae, Ulvales)	151.7 kDa/n.d.	Fe^2+^ chelation OH scavenging: IC_50_ > 1 mg/mL O_2_^−^ scavenging: IC_50_ = 22.1 μg/mL reducing power	[225]
**271**	*U. pertusa* (Chlorophyta, Ulvophyceae, Ulvales)	n.d./n.d.	Fe^2+^ chelation: 10% to 20% at 0.31–1.88 mg/mL OH scavenging: 3.3–37% at 0.25–1.52 mg/mL O_2_^−^ scavenging: IC_50_ = 9.17 μg/mL reducing power	[226]

AChE: acetylcholinesterase; BChE: butyrylcholinesterase; DPPH: 1,1-diphenyl-2-picrylhydrazyl free radical; GAE: gallic acid equivalents; GSH: glutathione; HO-1: heme oxygenase-1; LPS: lipopolysaccharide; MMP: matrix metalloproteinase; MPO: myeloperoxidase; n.d.: not determined; NO: nitric oxide; Nrf2: nuclear factor erythroid 2-related factor 2; OH: hydroxyl; O_2_^−^: superoxide anion; RC_0.5AU_: reducing capacity at 0.5 absorbance unit; ROS: reactive oxygen species; SOD: superoxide dismutase; TE: trolox equivalents.

**Table 7:** Miscellaneous compounds from macroalgae with antioxidant activity.

Compound	Isolation Source	Assay/Activity	Reference
**272**	*G. furcata* (Rhodophyta, Florideophyceae, Gigartinales)	DPPH scavenging: IC_50_ = 290.5 ± 1.5 μM ONOO^−^ scavenging: IC_50_ = 8.45 ± 0.46 μM AChE inhibition: IC_50_ = 94.4 ± 1.7 μM BChE inhibition: IC_50_ = 242.0 ± 4.8 μM	[75]
**273**	*G. furcata* (Rhodophyta, Florideophyceae, Gigartinales)	DPPH scavenging: IC_50_ > 274.4 ONOO^−^ scavenging: IC_50_ = 218.7 ± 1.5 μM AChE inhibition: IC_50_ = 31.2 ± 1.0 μM BChE inhibition: IC_50_ = 526.7 ± 6.1 μM	[75]
**274**	*G. furcata* (Rhodophyta, Florideophyceae, Gigartinales)	DPPH scavenging: IC_50_ > 195.0 μΜ ONOO^−^ scavenging: IC_50_ = 28.5 ± 0.0 μM AChE inhibition: IC_50_ = 33.9 ± 0.9 μM BChE inhibition: IC_50_ > 390.0 μM	[75]
**275**	*Cystoseira* sp. (Ochrophyta, Phaeophyceae, Fucales)	guglone-induced oxidative stress and intracellular ROS measurement in *Caenorhabditis elegans*	[141,232]
**276**	*G. furcata* (Rhodophyta, Florideophyceae, Gigartinales)	DPPH scavenging: IC_50_ > 179.6 μΜ ONOO^−^ scavenging: IC_50_ = 58.3 ± 0.3 μM AChE inhibition: IC_50_ = 44.9 ± 1.4 μM BChE inhibition: IC_50_ = 57.1 ± 2.7 μM	[75]
**277**	*G. furcata* (Rhodophyta, Florideophyceae, Gigartinales)	DPPH scavenging: IC_50_ > 165.3 μΜ ONOO^−^ scavenging: IC_50_ = 52.4 ± 0.2 μM AChE inhibition: IC_50_ = 38.1 ± 1.4 μM BChE inhibition: IC_50_ = 21.7 ± 1.1 μM	[75]
**278**	*L. undulata* (Rhodophyta, Florideophyceae, Ceramiales)	alkyl scavenging: IC_50_ = 45.0 ± 1.6 µM DPPH scavenging: IC_50_ = 27.1 ± 1.1 µM OH scavenging: IC_50_ = 22.8 ± 0.8 µM O_2_^−^ scavenging: IC_50_ = 33.5 ± 1.3 µM gene expression of enzymes GSH and SOD intracellular ROS levels (DCFH–DA) in RAW264.7 cells membrane protein oxidation MPO activity	[233]
**279**	*G. furcata* (Rhodophyta, Florideophyceae, Gigartinales)	DPPH scavenging: IC_50_ > 220.9 μΜ ONOO^−^ scavenging: IC_50_ = 206.6 ± 1.0 μM AChE inhibition: IC_50_ = 13.6 ± 0.5 μM BChE inhibition: IC_50_ = 420.1 ± 7.8 μM	[75]
**280**	*Kappaphycus alvarezii* (Rhodophyta, Florideophyceae, Gigartinales)	ABTS^+^ scavenging: IC_50_ = 3.63 ± 0.55 mM DPPH scavenging: IC_50_ = 3.53 ± 0.05 mM	[234]
**281**	*K. alvarezii* (Rhodophyta, Florideophyceae, Gigartinales)	ABTS^+^ scavenging: IC_50_ = 1.96 ± 0.51 mM DPPH scavenging: IC_50_ = 1.75 ± 0.20 mM	[234]
**282**	*Jania rubens* (Rhodophyta, Florideophyceae, Corallinales)	ABTS^+^ scavenging: IC_50_ = 1.48 mM DPPH scavenging: IC_50_ = 0.80 mM	[235]
**283**	*S. wightii* (Ochrophyta, Phaeophyceae, Fucales)	ABTS^+^ scavenging: IC_50_ = 2.89 ± 0.04 mM DPPH scavenging: IC_50_ = 2.44 ± 0.11 mM Fe^2+^ chelation: IC_50_ = 3.64 ± 0.08 mM	[236]
**284**	*S. wightii* (Ochrophyta, Phaeophyceae, Fucales)	ABTS^+^ scavenging: IC_50_ = 3.76 ± 0.08 mM DPPH scavenging: IC_50_ = 3.26 ± 0.04 mM Fe^2+^ chelation: IC_50_ = 4.65 ± 0.08 mM	[236]
**285**	*K. alvarezii* (Rhodophyta, Florideophyceae, Gigartinales)	ABTS^+^ scavenging: IC_50_ = 0.67 ± 0.25 mM DPPH scavenging: IC_50_ = 0.61 ± 0.06 mM	[234]
**286**	*Gracilaria opuntia* (Rhodophyta, Florideophyceae, Gracilariales	ABTS^+^ scavenging: IC_50_ = 0.50 mM DPPH scavenging: IC_50_ = 0.41 mM	[237]
**287**	*C. trinodis* (Ochrophyta, Phaeophyceae, Fucales) most probably as a contamination from *Laurencia* sp. (Rhodophyta, Florideophyceae, Ceramiales)	ABTS^+^ scavenging: 26.01 ± 0.01%	[135]
**288**	*K. alvarezii* (Rhodophyta, Florideophyceae, Gigartinales)	ABTS^+^ scavenging: IC_50_ = 1.30 ± 0.48 mM DPPH scavenging: IC_50_ = 0.97 ± 0.07 mM	[238]
**289**	*K. alvarezii* (Rhodophyta, Florideophyceae, Gigartinales)	ABTS^+^ scavenging: IC_50_ = 2.28 mM DPPH scavenging: IC_50_ = 2.02 mM	[235]
**290**	*K. alvarezii* (Rhodophyta, Florideophyceae, Gigartinales)	ABTS^+^ scavenging: IC_50_ = 1.42 mM DPPH scavenging: IC_50_ = 2.50 mM	[235]
**291**	*Spatoglossum variabile* (Ochrophyta, Phaeophyceae, Dictyotales)	O_2_^−^ scavenging: IC_50_ = 22.2 μM	[239]
**292**	*S. wightii* (Ochrophyta, Phaeophyceae, Fucales)	ABTS^+^ scavenging: IC_50_ = 1.28 ± 0.00 mM DPPH scavenging: IC_50_ = 1.05 ± 0.03 mM	[240]
**293**	*Hypnea musciformis* (Rhodophyta, Florideophyceae, Gigartinales)	DPPH scavenging: IC_50_ = 231.2 ± 2.0 μM Fe^2+^ chelation: IC_50_ = 667.9 ± 0.8 μM lipid peroxidation (TBARS): 1.34 ± 0.01 MDAEQ/kg at 0.1 μg/mL	[241]
**294**	*S. wightii* (Ochrophyta, Phaeophyceae, Fucales)	ABTS^+^ scavenging: IC_50_ = 1.81 ± 0.03 mM DPPH scavenging: IC_50_ = 1.2 ± 0.05 mM Fe^2+^ chelation: IC_50_ = 2.28 ± 0.03 mM	[236]
**295**	*T. conoides* (Ochrophyta, Phaeophyceae, Fucales)	ABTS^+^ scavenging: IC_50_ = 2.00 mM DPPH scavenging: IC_50_ = 1.71 mM	[242]
**296**	*T. conoides* (Ochrophyta, Phaeophyceae, Fucales)	ABTS^+^ scavenging: IC_50_ = 1.39 mM DPPH scavenging: IC_50_ = 1.29 mM	[242]
**297**	*H. musciformis* (Rhodophyta, Florideophyceae, Gigartinales)	DPPH scavenging: IC_50_ = 25.0 ± 0.5 μM Fe^2+^ chelation: IC_50_ = 350.7 ± 0.5 μM lipid peroxidation (TBARS): 0.88 ± 0.01 MDAEQ/kg at 0.1 μg/mL	[241]
**298**	*H. musciformis* (Rhodophyta, Florideophyceae, Gigartinales)	DPPH scavenging: IC_50_ = 322.4 ± 1.1 μM Fe^2+^ chelation: IC_50_ = 5115.3 ± 2.1 μM lipid peroxidation (TBARS): 0.76 ± 0.01 MDAEQ/kg at 0.1 μg/mL	[241]
**299**	*S. wightii* (Ochrophyta, Phaeophyceae, Fucales)	ABTS^+^ scavenging: IC_50_ = 0.81 ± 0.04 mM DPPH scavenging: IC_50_ = 0.64 ± 0.02 mM Fe^2+^ chelation: IC_50_ = 1.42 ± 0.02 mM	[236]
**300**	*S. wightii* (Ochrophyta, Phaeophyceae, Fucales)	ABTS^+^ scavenging: IC_50_ = 0.79 ± 0.03 mM DPPH scavenging: IC_50_ = 0.67 ± 0.03 mM	[240]
**301**	*T. conoides* (Ochrophyta, Phaeophyceae, Fucales)	ABTS^+^ scavenging: IC_50_ = 2.18 mM DPPH scavenging: IC_50_ = 1.95 mM	[242]

ABTS^+^: 2,2’-azino-bis (3-ethyl benzothiazoline-6-sulfonic acid) diammonium salt; AChE: acetylcholinesterase; BChE: butyrylcholinesterase; DPPH: 1,1-diphenyl-2-picrylhydrazyl free radical; IC_50_: half maximal inhibitory concentration; MDAEQ/kg: malondialdehyde equivalent compounds formed per kg sample; ONOO^−^: peroxynitrite; O_2_^−^: superoxide anion; TBARS: thiobarbituric acid reactive substances.

## Data Availability

Not applicable.
